# Error Awareness Can Occur in the Absence of an Error‐Related Negativity

**DOI:** 10.1111/psyp.70128

**Published:** 2025-10-07

**Authors:** Julia Dumsky, Martin E. Maier, Francesco Di Gregorio, Marco Steinhauser

**Affiliations:** ^1^ Department of Psychology Catholic University of Eichstätt‐Ingolstadt Eichstätt Germany; ^2^ Center for Studies and Research in Cognitive Neuroscience, Department of Psychology Alma Mater Studiorum – University of Bologna Cesena Italy

**Keywords:** error awareness, error positivity, error‐related negativity, event‐related potentials, metacognition

## Abstract

Errors in choice tasks lead to a cascade of error‐related brain activity in event‐related potentials. While the error‐related negativity (Ne/ERN) reflects an early error signal, the error positivity (Pe) has been attributed to the later emergence of error awareness. Previous work has shown that these two components can be dissociated using a target‐masking paradigm. In this modified flanker paradigm, an invisible‐target condition is realized in which errors are detectable even if the correct response is unknown. These errors have been shown to elicit a Pe without a Ne/ERN, demonstrating the independence of the two underlying systems. Here, we employed this paradigm to ask whether error awareness can emerge without a Ne/ERN. While performing the target‐masking paradigm, participants provided metacognitive judgments to indicate whether an error has occurred on each trial (i.e., error signaling). The majority of participants were able to report detectable errors in the invisible‐target condition. Crucially, this error signaling as well as a Pe was observable in the absence of a Ne/ERN. Our findings demonstrate that both error awareness (as indicated by successful error signaling) and the Pe do not depend on the early error signal reflected by the Ne/ERN and thus confirm the existence of two independent systems of error monitoring.

## Impact statement

Errors in choice tasks lead to an early error‐related negativity (Ne/ERN) and a later error positivity (Pe). While the Pe has been shown to be closely linked to error awareness, the relationship between the Ne/ERN and error awareness has remained elusive. Here, we demonstrate that error awareness is possible even in the absence of an Ne/ERN, suggesting that error awareness and the Ne/ERN rely on different neural mechanisms.

## 1 Introduction

Detecting errors is a crucial precondition for adaptive behavior. It has long been known that the human brain is equipped with an error monitoring system that registers errors and then initiates adjustments to attention and behavior to prevent errors in the future (Gehring et al. 2012; Ullsperger, Danielmeier, and Jocham 2014; Ullsperger, Fischer, et al. 2014). The most prominent neural correlate of this system is the error‐related negativity (ERN; Gehring et al. 1993) or error negativity (Ne; Falkenstein et al. 1991), reflecting an early error signal that is elicited almost immediately after an error. Another important aspect of error processing is the ability to become consciously aware of errors (Wessel et al. 2011). Decades ago, it has been shown that when humans commit errors in speeded perceptual choice tasks, they can indicate these errors with high reliability within the fraction of a second (Rabbitt 1966). Although research on the functional architecture of error awareness has made considerable progress in recent years, many fundamental questions are still unanswered. In the present study, we investigate to which extent error awareness relies on early error processing reflected by the Ne/ERN. More specifically, we ask whether error awareness can occur in errors in which the Ne/ERN is absent.

The Ne/ERN is a negativity with a maximum around 50 ms after an erroneous response at frontocentral electrodes and has its origin in the medial‐frontal cortex (Debener et al. 2005; Iannaccone et al. 2015; Ullsperger and von Cramon 2001). It has initially been suggested to represent a signal which indicates a mismatch between the actual and correct response (Fu et al. 2023; Scheffers and Coles 2000); whereas later theoretical accounts proposed that it is a representation of a postresponse conflict (Yeung et al. 2004) or a prediction error (Holroyd and Coles 2002). In all these accounts, this early component of error processing relies on a discrepancy between the expected correct response and the actual response and hence requires that a representation of this correct response is available. A second component, the error positivity (Pe; Falkenstein et al. 1991; Overbeek et al. 2005), occurs between 200 ms and 500 ms after the error and has a more posterior distribution on the scalp. The Pe has frequently been related to error awareness (Steinhauser and Yeung 2010; Ullsperger, Danielmeier, and Jocham 2014; Ullsperger, Fischer, et al. 2014) or the confidence about the correctness of a response (Boldt and Yeung 2015).

Error awareness can be measured by a metacognitive judgment called error signaling. After the primary task response, participants press a key to indicate whether an error has occurred or not. A frequent finding is that the Pe is larger on signaled errors than on unsignaled errors (e.g., Endrass et al. 2007; Kirschner et al. 2021; Nieuwenhuis et al. 2001; O'Connell et al. 2007; Porth et al. 2022; Steinhauser and Yeung 2010) or is observed only on signaled errors (e.g., Murphy et al. 2012). Moreover, the Pe has been shown to be generally larger if error signaling is required than if not (Grützmann et al. 2014). Based on findings like these, the idea has been formulated that the Pe amplitude represents the accumulated evidence for an error that eventually leads to error awareness as measured by error signaling (Desender et al. 2021; Steinhauser and Yeung 2010; Ullsperger, Danielmeier, and Jocham 2014; Ullsperger, Fischer, et al. 2014), and several studies provided direct evidence for this hypothesis (e.g., Steinhauser and Yeung 2010, 2012).

However, it remains unclear which role is played by the Ne/ERN for the emergence of error awareness and thus the ability to signal errors. A plausible assumption is that the early error signal represented by the Ne/ERN provides the evidence that is fed into evidence accumulation represented by the Pe and thus error awareness. For instance, Yeung et al. (2004) proposed that the Ne/ERN represents response conflict and that an error is signaled whenever this accumulated response conflict exceeds a criterion (see also Steinhauser et al. 2008). However, several findings speak against the idea that the Ne/ERN is the sole source of evidence for error awareness. First, the link between the Ne/ERN and error awareness is much weaker than that between the Pe and error awareness (for a review, see Wessel 2012). Whereas some studies reported a larger Ne/ERN for signaled errors than for unsignaled errors (e.g., Scheffers and Coles 2000; Wessel et al. 2011), others did not find such a relation (e.g., Endrass et al. 2007; Hughes and Yeung 2011; Nieuwenhuis et al. 2001; Porth et al. 2022). Second, many studies demonstrated that experimental manipulations had different effects on the Ne/ERN and Pe (e.g., Charles et al. 2013; Hughes and Yeung 2011; Maier et al. 2015; Steinhauser and Yeung 2010). These findings do not rule out that error awareness can be based on early error signals reflected by the Ne/ERN. But they imply that the Ne/ERN is not the only source of evidence that drives error awareness. In this vein, Wessel et al. (2011) proposed that error awareness might rely on evidence provided by perceptual, cognitive, and autonomous processes.

While the link between the Ne/ERN and error awareness appears to be weaker than initially assumed, results from another study even raise the possibility that error awareness can emerge without an Ne/ERN at all. In the study of Di Gregorio et al. (2018), participants performed a three‐choice flanker task, in which participants had to classify a central target letter while ignoring two laterally presented identical flanker letters. The target was always associated with a different response than the flankers, and participants were explicitly instructed that a response to the flanker letter is always an error. Crucially, the target letter was masked with variable stimulus‐mask intervals (SMI). In most trials, the SMI was sufficiently long for the target to be easily visible. But in one third of trials, only the mask was presented, and no target was visible at all. This invisible‐target condition created a scenario in which an error was detectable (whenever participants accidentally responded to the flankers) but the correct response remained unknown (because the target was fully masked). In this condition, Di Gregorio et al. (2018) observed a Pe in the absence of an Ne/ERN. This demonstrated that the Ne/ERN and Pe represent independent and dissociable systems of error monitoring: a system associated with the Ne/ERN that elicits early error signals and relies on a representation of the correct response and another system associated with the Pe that forms the basis of error awareness and can rely on other types of evidence (such as the knowledge that a response to the flanker must be an error).

Demonstrating that a Pe can occur in the absence of an Ne/ERN (Di Gregorio et al. 2018) has fundamental implications for the functional architecture of error monitoring. It shows that the Ne/ERN and Pe are not two stages of a single process (e.g., Yeung et al. 2004; Ullsperger, Fischer, et al. 2014) but rather form two independent systems (Charles et al. 2013; Maier et al. 2015). Crucially, as the Pe has been suggested to represent the evidence accumulation process that leads to error awareness (Steinhauser and Yeung 2010), this result further suggests that error awareness can also emerge in the absence of an Ne/ERN. However, direct evidence for this conjecture has not been provided because the study of Di Gregorio et al. (2018) did not include an error signaling task. In the present study, we combined the target‐masking paradigm from Di Gregorio et al. (2018) with an error signaling task. Participants had to indicate after each response whether this response was an error, a correct response, or whether they do not know. The latter category was introduced because some errors in this paradigm are objectively undetectable. Our main goal was to test whether objectively detectable and signaled errors in the invisible‐target condition lead to a Pe in the absence of an Ne/ERN. This would demonstrate that error awareness without an Ne/ERN is possible and would replicate the findings of Di Gregorio et al. (2018) regarding the dissociation of the Pe and Ne/ERN.

## 2 Method

### 2.1 Participants

We aimed to obtain the same sample size (*n* = 20) as Di Gregorio et al. (2018). This sample size implies a power of > 90% to detect within‐subject effects with a Cohen's *d* > 0.79, which was the smallest effect size of a significant contrast (correct vs. error) reflecting an Ne/ERN or Pe in Di Gregorio et al. (2018). However, because not all participants showed a sufficient detection performance in the invisible‐target condition, we had to collect more data to obtain at least 20 participants for the main analysis. Eventually, thirty‐three participants participated in the study (self‐reported gender: 25 female, 7 male, 1 nonbinary). All participants were recruited at the Catholic University of Eichstätt‐Ingolstadt, had normal or corrected‐to‐normal vision, had an age between 19 and 38 years (*M* = 20.9, SD = 2.57) and received either course credits or 8 Euro per hour. The study was approved by the ethics committee of the university, and informed consent was obtained from all participants.

### 2.2 Task and Stimuli

Stimuli were presented using Presentation software (Neurobehavioral Systems, Albany, CA) on a 21‐in. screen with a resolution of 1280 × 1024 and a refresh rate of 60 Hz. Viewing distance was approximately 70 cm. Each trial comprised a primary task and a secondary task. The *primary task* required participants to classify a target letter by pressing the ‘A’, ‘S’, or ‘D’ key of a standard computer keyboard with the ring finger, middle finger, and index finger, respectively, of their left hand. The primary task stimuli were horizontal strings of seven white letters presented on a black background. They consisted of a central target letter and three identical flanker letters on each side of the central target. The letters P, W, M, V, X, or K were used, each with a height of 0.41° visual angle and a width of 0.25° visual angle. The same pairs of letters (‘PW’, ‘MV’ and ‘XK’) were assigned to one of the response buttons, for example, ‘P’ to the button ‘A’, ‘MV’ to the button ‘S’, and ‘XK’ to the button ‘D’. The assignment of these pairs of letters to the three response buttons was additionally varied across participants. This resulted in six different combinations of the stimulus–response mappings. All stimuli were incongruent, that is, target and flanker letters were always assigned to different responses. This resulted in 24 possible stimuli. Six different feature masks of the same size as a letter were created by randomly rearranging features of the original letter stimuli. The *secondary task* required participants to judge the correctness of their primary task response by pressing one of three keys with their right hand: (a) ‘H’ key with the index finger if the previous response was correct, (b) the ‘J’ key with their middle finger if the previous response was an error, or (c) the ‘K’ key with the ring finger if they did not know whether the previous response was correct or not (“unsure”).

**FIGURE 1:**
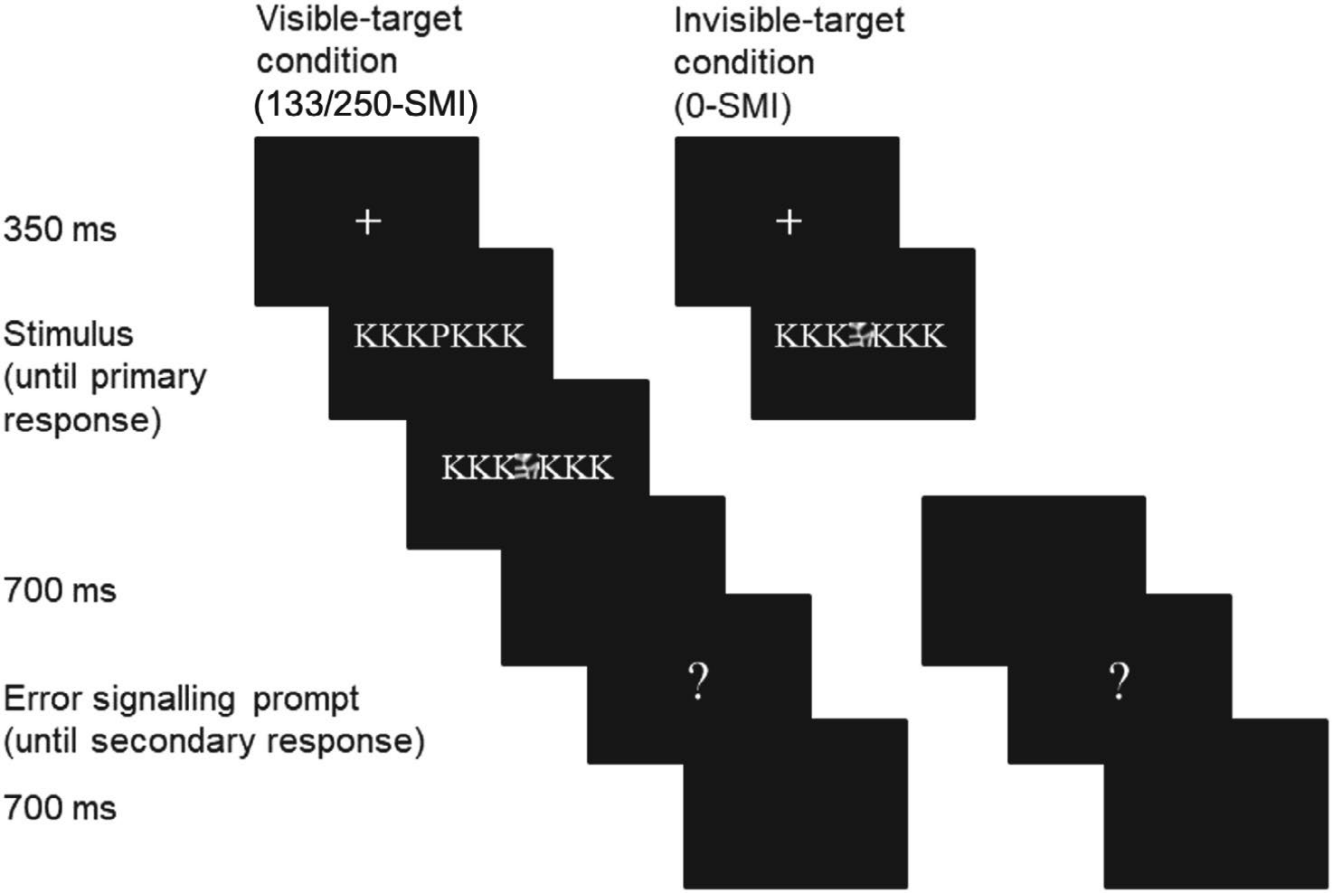
Typical trial sequence. Participants had to respond to the target letter while flanker letters had to be ignored. In the visible‐target condition, the target letter was masked after 133 or 250 ms. In the invisible‐target condition, only the mask was presented but no target. After the response, a prompt appeared, and participants had to indicate whether the response was correct, incorrect, or whether they do not know. SMI = stimulus‐mask interval.

An exemplary trial is depicted in Figure 1. At the beginning of each trial, a fixation cross was presented for 350 ms, followed by the primary task stimulus. One of the six masks replaced the central target after a stimulus‐masking interval (SMI) of 0 ms, 133.3 ms, or 250 ms. Whereas participants could see a target in the 133‐SMI and 250‐SMI trials (the *visible‐target condition*), no target was presented at all in the 0‐SMI condition (the *invisible‐target condition*, Figure 1B). Mask and flankers were presented on the screen until the participant provided the primary task response. After the response, a black screen was presented for 700 ms followed by a white question mark, which remained on the screen until the secondary task response was made. At the end of each trial, another black screen was presented for 700 ms again.

Each block consisted of 72 trials, in which each of the possible 24 stimuli was combined with each of the three possible SMIs. Stimuli and conditions were presented in pseudo‐randomized order (without any constraints) within blocks. The experiment was conducted over a two‐day period, with a maximal interval of 48 h in between. The first day was used for practicing the primary as well as the secondary task. In the first two practice blocks, the participants practiced only the primary task. Then, the secondary task was introduced, and the participants had to complete ten blocks with both tasks. During the whole practice session, the participants could look at a paper sheet displaying the rules for the primary and secondary task for support. During all practice blocks, a deadline was implemented to train the participants to provide their primary response within a certain time interval and in this way to commit a sufficient number of erroneous trials. If this deadline was exceeded, automatic feedback about the latency was presented at the center of the screen, which urged the participant to respond faster (“SCHNELLER”). Each participant started with the same deadline of 500 ms. Afterwards, it was adapted from trial to trial in the following way (see Steinhauser and Yeung 2010, for a similar procedure): if the primary task response in the previous trial was incorrect, the deadline in the current trial was increased by 100 ms. If the response was correct, the deadline was decreased by 20 ms (but could never fall below 100 ms). A 5:1 ratio was applied to achieve an approximate error rate of 15% (Kaernbach 1991). This trial‐to‐trial adaptation was only applied on 133‐SMI and 250‐SMI trials, while the deadline remained unchanged (i.e., the same as after the previous trial) on 0‐SMI trials. Moreover, if the previous trial was a 0‐SMI trial, the deadline was adapted based on the last 133‐SMI or 250‐SMI trial.

On the second day, the test session was conducted. The participants first completed two practice blocks with 48 trials each (including the deadline procedure). Then, twelve test blocks were conducted in which no feedback was provided. Instead, the participants were instructed at the beginning of a block to respond faster whenever the number of errors in the visible‐target condition fell below eight (16.7%). At the beginning of each session, participants were instructed about the tasks. Specifically, they received the information that (a) they should guess if the target letter could not be identified as sometimes the target would be very difficult to see, and (b) responding to the flankers would always be an error. The whole test session lasted approximately 1.5 h, and the participant could take short breaks between blocks whenever necessary.

### 2.3 EEG Data Acquisition

Only during the test session, the electroencephalogram was recorded using a BIOSEMI Active‐Two system (BioSemi, Amsterdam, The Netherlands) with 64 Ag‐AgCl electrodes in a 10–10 montage from channels Fp1, AF7, AF3, F1, F3, F5, F7, F17, FC5, FC3, FC1, C1, C3, C5, T7, TP7, CP5, CP3, CP1, P1, P3, P5, P7, P9, PO7, PO3, O1, Iz, Oz, POz, Pz, CPz, Fpz, Fp2, AF8, AF4, AFz, Fz, F2, F4, F6, F8, FT8, FC6, FC4, FC2, FCz, Cz, C2, C4, C6, T8, TP8, CP6, CP4, CP2, P2, P4, P6, P8, P10, PO8, PO4, O2, as well as the left and right mastoid. The CMS (Common Mode Sense) and DRL (Driven Right Leg) electrodes were used as reference and ground electrodes. The vertical and horizontal electrooculogram (EOG) was recorded with BioSemi FLAT Active electrodes positioned over and under the right eye as well as on the outer canthi of both eyes. EEG and EOG were continuously recorded at a sampling rate of 512 Hz. No online filtering was applied, and all electrodes were off‐line re‐referenced to the average reference.

### 2.4 Data Analysis

Only data from the test blocks were analyzed; the first three trials in each block were removed from all analyses. A Greenhouse–Geisser correction was conducted (Greenhouse and Geisser 1959) where necessary, and corrected *p*‐values but uncorrected degrees of freedom were reported in this case.

### 2.5 Response Categories

Each trial was assigned to a response category depending on the primary response (for an example, see Figure 1). In the visible‐target condition (133/250‐SMI), there were three possible response categories: (1) A *correct response* resulted if the response matched the target response. (2) A *flanker error* resulted if the response matched the flanker response. (3) A *nonflanker error* resulted if the response matched neither the target nor the flanker response. In the invisible‐target condition (0‐SMI), there were two possible response categories: (1) A *flanker error* resulted if the response matched the flanker response. (2) A *nonflanker guess* resulted if the response did not match the flanker response. Depending on the secondary response, trials were further classified as *“correct”*, *“error”*, or *“unsure”* (note that quotation marks are used to indicate the response categories of the secondary response).

### 2.6 Behavioral Data

The statistical analyses of the behavioral data were conducted with R Studio version 4.0.3 for Windows (R Core Team, released 2020, Vienna, Austria). The time between stimulus onset and the primary response was defined as response time (RT). If the RT deviated more than three standard deviations from the condition mean, the corresponding trial was excluded from the analyses. Overall, 0.29% of trials (SE = 0.004%) were excluded in this way. For frequency data, an arcsine transformation was performed to stabilize variances before statistical testing (Winer et al. 1971).

We first analyzed behavioral data to investigate whether reliable task performance and error signaling was possible in our paradigm. For the *visible‐target condition*, paired *t*‐tests were used to compare error rates and proportions of flanker errors (among all errors) between 133‐SMI and 250‐SMI conditions. Mean RTs were subjected to a two‐way repeated‐measures ANOVA with the variables SMI (133‐SMI, 250‐SMI) and correctness (correct, errors). Mean error RTs were further analyzed using a two‐way repeated‐measures ANOVA with the variables SMI and error type (flanker error, nonflanker error). To investigate whether reliable error signaling was achieved in the visible‐target condition, the rate of detected errors and the rate of errors that were classified as “unsure” was subjected to a two‐way ANOVA with the variables SMI and error type. The rate of false alarms and the rate of correct trials classified as unsure were analyzed using paired *t*‐tests to compare between 133‐SMI and 250‐SMI conditions. For the *invisible‐target condition*, primary task performance was analyzed by comparing mean RTs between flanker errors and nonflanker guesses using a paired *t*‐test. To see whether participants were able to detect errors by taking the flanker into account, we compared the rate of detected errors and the rate of errors that were classified as “unsure” between the two trial types (flanker errors, nonflanker guesses).

### 2.7 EEG Data

EEG data were analyzed using custom routines based on EEGLab (Delorme and Makeig 2004) implemented in MatLab R2013b (The Mathworks, Natick, MA). Preprocessing was the same as in Di Gregorio et al. (2018) unless otherwise stated. EEG data were first low‐pass filtered (40 Hz) with a least‐square FIR filter (−6 dB cutoff) and then high‐pass filtered (0.1 Hz). Epochs were extracted from 200 ms before and 600 ms after the primary response. Baseline correction was applied based on the interval [−100 ms; 0 ms], which was a different baseline than in Di Gregorio et al. (2018) (in which [−150 ms; −50 ms] was used). This was done to properly align correct and error trials at the onset of the Ne/ERN to prevent overestimation or underestimation of error‐related activity. Channels were interpolated using spherical spline interpolation whenever they met the joint probability criterion (threshold 5) or the kurtosis criterion (threshold 10) in EEGLAB's pop_rejchan function. In this way, 4.22 (SE = 0.21) channels were interpolated on average. Epochs were excluded if the amplitude exceeded a threshold of ±200 μV at any electrode (except at the frontal electrodes as blinks were removed at a later stage) or if the joint probability deviated more than 5 standard deviations from the epoch mean (according to EEGLAB's pop_jointprob function). On average, 4.63% of trials (SE = 0.24%) were rejected in this way. In the condition with the smallest trial number (nonflanker errors in the 250‐SMI condition), an average of 20 trials (SE = 3.0) remained in the analysis. An independent component analysis (Delorme and Makeig 2004) was computed, and independent components with blink artifacts were removed using CORRMAP (Campos Viola et al. 2009). Finally, epochs were averaged separately for each condition and participant. The Ne/ERN was quantified as mean amplitude in the time window [0 ms; 100 ms] at a frontocentral electrode cluster including electrodes Cz, FC1, FCz, FC2, and Fz. The Pe was quantified as mean amplitude in the time window [200 ms; 500 ms] at a posterior electrode cluster including electrodes POz, P1, Pz, P2, and CPz.

Because waveforms from the frontocentral cluster suggested that there might be an earlier Ne/ERN peak with a more variable latency in the invisible‐target condition, we additionally ran a control analysis with an alternative quantification method. The so‐called adaptive mean measure (Clayson et al. 2013) requires, first, identifying the largest negative peak in the difference wave between errors and correct trials separately for each participant, and second, analyzing mean amplitudes around these individual peak latencies. This quantification method is more sensitive than standard methods if peak latencies vary across participants. It works particularly well if a large Ne/ERN is present and thus the largest negative peak is indeed due to an Ne/ERN. However, in a scenario like ours in which an Ne/ERN is possibly absent and differences between errors and corrects might reflect noise, then the method could artificially induce an Ne/ERN‐like negativity because there is necessarily always a negative peak in noisy data. We therefore modified this method to make it applicable to a scenario in which the goal of the analysis is to decide whether an Ne/ERN is present at all: Instead of identifying the largest negative peak, we identified the largest absolute peak (irrespective of whether it is positive or negative). If there is no Ne/ERN, then the average of participants with positive and negative maxima should be zero (which is the null hypothesis). If an Ne/ERN is present, then this mean should be significantly negative. As the data suggest that an Ne/ERN peak in the invisible‐target condition might occur earlier than the original time window, we used a larger and earlier time window (−50 ms to 100 ms) and consequently an earlier baseline (−150 to −50 ms) for this analysis. Mean amplitudes were analyzed in a time window of 100 ms centered around the respective peaks.

In all reported analyses, we included only correctly classified errors and correct trials as well as nonflanker guesses for which participants indicated to be unsure. We report only analyses of the good detector group, as only these participants had sufficient numbers of detected errors (see Supporting Information). In the *visible‐target condition*, the Ne/ERN and Pe (defined as the amplitude difference between correct responses and errors in the respective time window) were subjected to a two‐way repeated‐measures ANOVA with the variables SMI (133‐SMI, 250‐SMI) and error type (flanker error, nonflanker error). Paired *t*‐tests were used for follow‐up analyses. While these analyses should demonstrate how an Ne/ERN and Pe look like if the target is visible, analyses of the *invisible target condition* serve to determine whether an Ne/ERN and/or Pe is obtained if the target is invisible and the correct response is unknown. Only for the Ne/ERN analyses in this part, we additionally used the modified adaptive mean measure described above. We first tested for the presence of an Ne/ERN and Pe by directly comparing flanker errors and nonflanker guesses against the correct baseline from the 133‐SMI condition using paired *t*‐tests (see results section for a discussion of the choice of this baseline). Moreover, paired *t*‐tests were also used to test for differences between flanker errors and nonflanker guesses. In a final set of analyses, the Ne/ERN and Pe for flanker errors were first subjected to separate one‐way repeated‐measures ANOVAs with the variable SMI (0‐SMI, 133‐SMI, 250‐SMI). Then the pattern of Ne/ERNs and Pes on flanker errors was directly compared using a two‐way repeated‐measures ANOVA with the variables component (Ne/ERN, Pe) and SMI (0, 133, 250) to statistically establish the dissociation between the two components.

## 3 Results

A crucial goal of this study was to investigate whether the Ne/ERN was absent for detected flanker errors in the invisible‐target condition, and thus, whether error signaling as a behavioral correlate of error awareness is possible without an Ne/ERN. However, participants turned out to differ with respect to whether they consistently detected these errors, and visual inspection of the distribution of the rate of detected flanker errors suggested a bimodal distribution (see Supporting Information for more information). We therefore split our sample into two subgroups: a group of good detectors (*n* = 21) with a detection rate of more than 40% and a group of bad detectors (*n* = 12) with a detection rate of less than 40%. The 40% criterion was selected as it (a) separated the two groups based on their detection rate, and (b) ensured a sufficient number of detected flanker errors for EEG analysis for each participant in this group (> = 35 trials, see Supporting Information). In the following, only the results for the good detectors group are presented; however, a comparison of the two groups is provided in the Supporting Information.

### 3.1 Behavioral Data

#### 3.1.1 Visible‐Target Condition (133/250‐SMI)

We first analyzed the behavioral data for the visible‐target condition (133‐SMI, 250‐SMI) to investigate whether participants reliably detected errors in this condition. Primary task performance (see Table 1) showed differences between trials with SMIs of 133 ms and 250 ms. Error rates were higher for the 133‐SMI condition than for the 250‐SMI condition; *t*(20) = 3.56, *p* = 0.002, *d* = 0.778. Also, RTs were higher in the 133‐SMI than in the 250‐SMI condition; *F*(1, 20) = 13.7, *p* = 0.001, *η*_*p*_^2^ = 0.407, and were higher for correct than for error trials; *F*(1, 20) = 11.0, *p* = 0.002, *η*_*p*_^2^ = 0.355. Next, we differentiated between flanker errors and nonflanker errors. The relative frequency of flanker errors among all errors was 57% (SE = 1.9%) and did not differ between SMI conditions; *t*(20) = 0.22, *p* = 0.832, *d* = 0.048. Regarding error RTs, there was neither a significant difference between error types; *F*(1, 20) = 0.61, *p* = 0.445, *η*_*p*_^2^ = 0.030, nor between SMI conditions; *F*(1, 20) = 2.35, *p* = 0.141, *η*_*p*_^2^ = 0.105.

**TABLE 1:** Primary task performance.

Conditions	Error rates (%)	Prop. FE (%)	RT correct (ms)	RT FE (ms)	RT NFE/G (ms)
250‐SMI	16.0 (±1.7)	57.2 (±2.3)	537 (±17)	531 (±33)	523 (±31)
133‐SMI	22.5 (±1.6)	56.8 (±1.5)	564 (±18)	551 (±34)	537 (±26)
0‐SMI	—	31.4 (±1.4)	—	513 (±17)	549 (±23)

*Note:* NFE/G refers to NFE in the 250‐SMI and 133‐SMI conditions but NFG in the 0‐SMI condition. Within‐participants standard errors of the mean are provided in parentheses. Abbreviations: FE, flanker error; ms, milliseconds; NFE, nonflanker error; NFG, nonflanker guess; Prop, proportion; RT, response time; SMI, stimulus‐masking interval.

The detection rates revealed that participants were able to reliably detect errors in the visible‐target condition. 85% (SE = 3.3%) of errors were detected, which is comparable with previous findings with unmasked stimuli (Steinhauser et al. 2008). The rate of detected errors did not differ between error types, *F*(1, 20) = 2.38, *p* = 0.138, *η*_*p*_^2^ = 0.106, but was higher in the 250‐SMI condition (89%, SE = 3.1%) than in the 133‐SMI condition (81.1%, SE = 3.4%), *F*(1, 20) = 14.5, *p* = 0.001, *η*_*p*_^2^ = 0.420. In 8.7% (SE = 1.5%) of errors, participants were unsure whether an error had occurred or not, and this rate was higher for the 133‐SMI condition (*M* = 11.2%, SE = 2.1%) than for the 250‐SMI condition (*M* = 6.3%, SE = 1.9%), *F*(1, 20) = 8.43, *p* = 0.009, *η*_*p*_^2^ = 0.297, but did not differ across error types, *F*(1, 20) = 0.11, *p* = 0.745, *η*_*p*_^2^ = 0.006. The rate of correct trials that were falsely judged as “error” (false alarm rate) was low (0.53%, SE = 0.21%) and did not differ between SMI conditions, *t*(20) = 1.10, *p* = 0.285, *d* = 0.250, and the same held for the rate of correct trials judged as “unsure” (*M* = 0.71%, SE = 0.22%), *t*(20) = 1.43, *p* = 0.167, *d* = 0.312.

Taken together, we obtained considerable effects of the masking interval in almost all behavioral parameters. The 133‐SMI condition was associated not only with impaired error awareness but also with impaired primary task performance; an effect that was not found in our previous study (Di Gregorio et al. 2018).

#### 3.1.2 Invisible‐Target Condition (0‐SMI)

A central goal of the present study was to investigate whether error awareness is possible for flanker errors in the invisible‐target condition, that is, for errors for which we expected a Pe but no Ne/ERN. Because a correct response was not possible in this condition, participants could either commit a flanker error (because responses to the flankers were always errors) or a nonflanker guess. The rate of flanker errors (among all responses) in the invisible‐target condition was 31.4% (SE = 1.4%), and flanker errors had a lower RT than nonflanker guesses (Table 1); *t*(20) = 5.73, *p* < 0.001, *d* = 1.250.

Crucially, a substantial proportion of flanker errors in the invisible‐target condition was detected (81.6%, SE = 4.3%). This detection rate was higher than that for nonflanker guesses (2.9%, SE = 0.7) for which errors were not objectively detectable; *t*(20) = 16.1, *p* < 0.001, *d* = 3.513, but was not significantly different from the averaged detection rate for flanker errors in the visible‐target conditions (84.0%, SE = 3.1%); *t*(20) = 0.44, *p* = 0.667, *d* = 0.095. As one would expect, the rate of trials classified as “unsure” in turn was higher for nonflanker guesses (93.0%, SE = 1.7%) than for flanker errors (17.7%, SE = 4.2%); *t*(20) = 13.4, *p* < 0.001, *d* = 2.928. Finally, for the rate of trials classified as “correct”, no significant difference between flanker errors (0.8%, SE = 0.3%) and nonflanker guesses (4.2%, SE = 1.6%) was observed; *t*(20) = 1.98, *p* = 0.062, *d* = 0.095.

Taken together, the participants showed a pattern of error awareness that roughly corresponded to the objective probability of errors in the two trial types. Flanker errors were classified as “error” with a similar rate as flanker errors in the visible‐target condition. In contrast, nonflanker guesses were predominantly classified as “unsure”. Please note that this pattern differs from that in the bad detectors group; a detailed comparison of the two groups is provided in the Supporting Information.

### 3.2 ERP Data

We next analyzed ERP data to investigate whether the results from Di Gregorio et al. (2018) can be replicated for detected errors in the invisible‐target condition; and thus, whether error awareness can occur in the absence of a Ne/ERN. In all analyses, we included only correctly classified errors and correct trials as well as nonflanker guesses for which participants indicated to be unsure.

#### 3.2.1 Visible‐Target Conditions (133/250 **SMI** ) in Good Detectors

**FIGURE 2:**
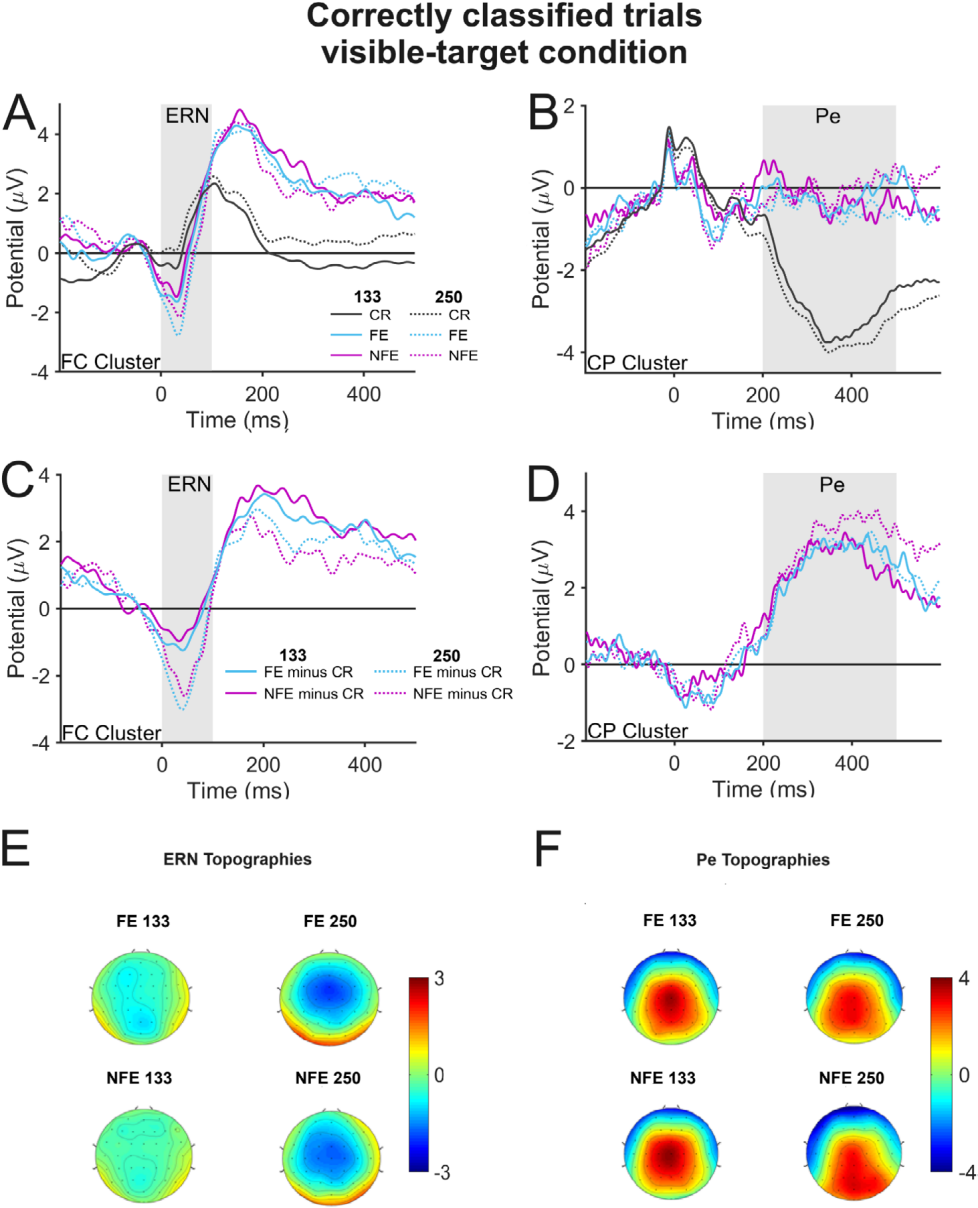
Error‐related negativity (Ne/ERN; frontocentral cluster in left column) and error positivity (Pe; centroparietal cluster in right column) in the visible‐target (133/250‐SMI) conditions. Only correctly classified trials of the good detectors group were considered. (A, B) Waveforms from all response types. (C, D) Difference waves for flanker errors (minus corrects) and nonflanker errors (minus corrects). (E, F) Topographies of the difference waves in the time range of the Ne/ERN and Pe. Gray areas indicated the time range of the Ne/ERN and Pe in each graph. CP, centroparietal; CR, correct response; FC, frontocentral; FE, flanker error; NFE, nonflanker error; SMI, stimulus‐mask interval.

We first analyzed the 250‐SMI and 133‐SMI conditions to demonstrate that a typical Ne/ERN and Pe can be shown in our paradigm. To visualize the Ne/ERN, waveforms for all possible response types (correct, flanker error, nonflanker error) for the frontocentral electrode cluster are shown in Figure 2A, and waveforms and topographies of the difference between errors and corrects are depicted in Figure 2C,E, respectively. Both error types show negative deflections relative to correct trials in both SMI conditions, and these negativities were maximal at frontocentral electrodes, thus demonstrating a typical Ne/ERN. The ANOVA on the difference between errors and correct trials revealed a significant effect of SMI, *F*(1, 20) = 9.15, *p* = 0.007, *η*_*p*_^2^ = 0.314, indicating a larger Ne/ERN for the 250‐SMI condition (*M =* −1.75 μV, SE = 0.89 μV) than for the 133‐SMI condition (*M =* −0.55 μV, SE = 0.62 μV). However, there was no main effect for error type, *F*(1, 20) = 1.3, *p* = 0.268, *η*_*p*_^2^ = 0.061, and no significant interaction, *F*(1, 20) < 0.01, *p* > 0.999, *η*_*p*_^2^ < 0.001. Collapsed across SMIs, the difference between errors and corrects in the time window of the Ne/ERN reached significance for flanker errors (*M* = −1.25 μV, SE = 0.19 μV), *t*(20) = 6.55, *p* < 0.001, *d* = 1.429, as well as for nonflanker errors (*M* = −1.08 μV, SE = 0.26 μV), *t*(20) = 4.13, *p* < 0.001, *d* = 0.901. For correct trials, the waveform in the time range of the Ne/ERN showed a more negative mean amplitude for the 133‐SMI condition (*M* = 0.62 μV, SE = 0.18 μV) than for the 250‐SMI condition (*M* = 1.09 μV, SE = 0.18 μV), which, however, reached only marginal significance, *t*(20) = 1.84, *p* = 0.080, *d* = 0.402.

Figure 2B,D,F show the same waveforms, difference waves, and topographies for the centroparietal electrode cluster to visualize the Pe. The amplitudes of both error types for both SMIs are clearly more positive than those of the corresponding correct trials, and these positivities have a centroparietal distribution, thus indicating a Pe. The ANOVA on the difference between errors and corrects revealed no significant main effects of error type, *F*(1, 20) = 0.68, *p* = 0.419, *η*_*p*_^2^ = 0.033, and SMI, *F*(1, 20) = 0.60, *p* = 0.449, *η*_*p*_^2^ = 0.029, and no significant interaction, *F*(1, 20) = 0.79, *p* = 0.385, *η*_*p*_^2^ = 0.038. Collapsed across SMIs, this difference reached significance for flanker errors (*M* = 2.75 μV, SE = 0.34 μV, *t*(20) = 8.05, *p* < 0.001, *d* = 1.757) as well as for the nonflanker errors (*M* = 2.92 μV, SE = 0.42 μV, *t*(20) = 4.13, *p* < 0.001, *d* = 0.901). Moreover, there was no significant difference in the time range of the Pe between correct trials in the two SMI conditions, *t*(20) = 1.35, *p* = 0.193, *d* = 0.295.

In sum, there was a highly significant Ne/ERN and Pe for both error types and SMI conditions. However, as for the behavioral data, we obtained differences between the two SMI conditions that were not observed in our previous study (Di Gregorio et al. 2018). There was a reduced Ne/ERN for the 133‐SMI condition as well as a trend toward different amplitudes for correct waveforms in the time range of the Ne/ERN. It appears that the shorter masking interval impaired error processing relative to the longer masking interval, and that the mask had also an effect on correct trials. While effects like these have previously been reported (e.g., Charles et al. 2013), they imply that we cannot collapse across the two conditions in our analyses.

#### 3.2.2 Invisible‐Target Condition (0‐ **SMI** ) in Good Detectors

Di Gregorio et al. (2018) found that errors in the invisible‐target condition were associated with a Pe even though no Ne/ERN was observed. Here, we asked whether we can replicate this result when only correctly classified trials (i.e., flanker errors classified as “errors” and nonflanker guesses classified as “unsure”) are included, thus demonstrating that error awareness is possible in the absence of an Ne/ERN. As in our previous study, we had to deal with the problem that no correct trials are available in the invisible‐target condition that can be used as a baseline for quantifying the Ne/ERN and Pe. Again, we applied two strategies to determine whether an Ne/ERN or Pe was present: First, we used correct trials from the visible‐target condition as a baseline. Because the 133‐SMI and 250‐SMI conditions differed in several aspects of the data including the waveform of correct trials, we decided to use only the 133‐SMI condition for which the masking interval was closer to that of the invisible‐target condition. Second, we compared flanker errors and nonflanker guesses in the invisible‐target condition. For flanker errors, the stimulus provides more objective information that an error has occurred, which should lead to stronger error signals and thus a larger Pe and also a larger Ne/ERN if our hypothesis is incorrect and an Ne/ERN is observed in this condition.

**FIGURE 3:**
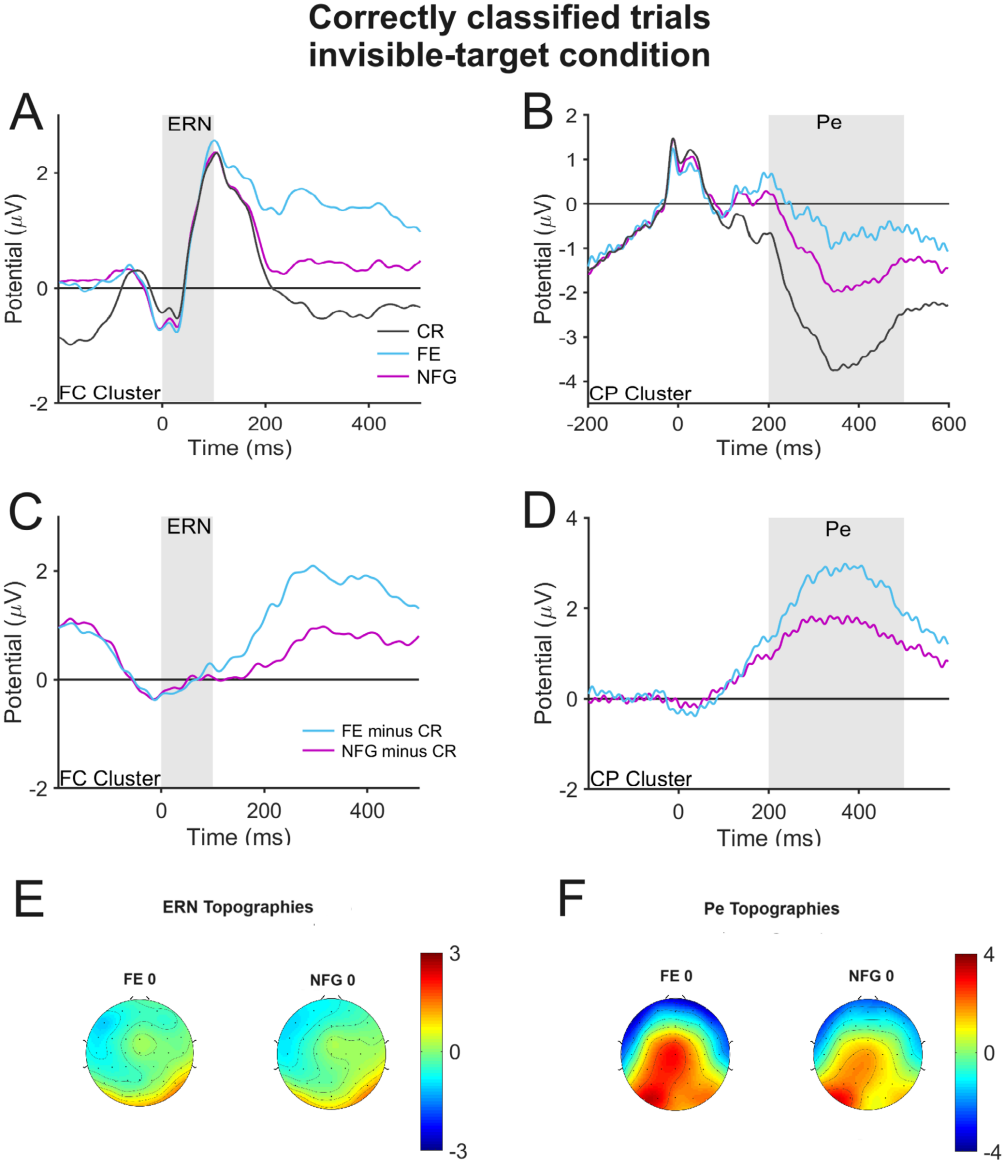
Error‐related negativity (Ne/ERN; frontocentral cluster in left column) and error positivity (Pe; centroparietal cluster in right column) in the invisible‐target (0‐SMI) condition. Only correctly classified trials of the good detectors group were considered. Data for correct responses were taken from the 133‐SMI condition. (A, B) Waveforms from all response types. (C, D) Difference waves for flanker errors (minus corrects) and nonflanker guesses (minus corrects). (E, F) Topographies of the difference waves in the time range of the Ne/ERN and Pe. Gray areas indicated the time range of the Ne/ERN and Pe in each graph. CP, centroparietal; CR, correct response; FC, frontocentral; FE, flanker error; NFG, nonflanker guess; SMI, stimulus‐mask interval.

To investigate whether a Ne/ERN is observable in the invisible‐target condition, we inspected waveforms and topographies from the frontocentral electrode cluster in Figure 3A,C. It reveals almost identical waveforms for all trial types in the time range of the Ne/ERN, suggesting that there is neither a negativity relative to the correct baseline nor a larger negativity for flanker errors than for nonflanker guesses. Also, the scalp topographies (Figure 3E) provided no evidence for a frontocentral negativity that could be interpreted as a Ne/ERN. These observations received support from statistical analyses. Neither the difference between flanker errors and correct trials (*M =* −0.08 μV, SE = 0.24 μV) reached significance; *t*(20) = 0.33, *p* = 0.749, *d* = 0.072, nor the difference between nonflanker guesses and correct trials (*M =* −0.07 μV, SE = 0.27 μV), *t*(20) = 0.24, *p* = 0.814, *d* = 0.052, and also waveforms for flanker errors and nonflanker guesses did not differ significantly; *t*(20) = 0.10, *p* = 0.923, *d* = 0.022. For completeness, we repeated this analysis with correct trials of the 250‐SMI condition. Correct trials in this condition differed significantly from flanker errors (*M =* −0.55 μV, SE = 0.20 μV), *t*(20) = 2.79, *p* = 0.011, *d* = 0.609, as well as nonflanker guesses (*M = −*0.53 μV, SE = 0.21 μV), *t*(20) = 2.52, *p* = 0.020, *d* = 0.550. However, these significant differences are most likely due to the difference between correct trials of the 133‐SMI and 250‐SMI reported above.

A closer inspection of Figure 3C shows that the difference wave peaks earlier in the invisible‐target condition than in the visible‐target condition. Moreover, Figure 3A suggests that the peak in the averaged waveforms of errors is not clearly identifiable, which could point to variability in the latency of the alleged Ne/ERN across participants. We therefore ran a control analysis in which we used a modified adaptive mean measure (Clayson et al. 2013) that can deal with variable peak latencies and applied this to a larger and earlier time window (−50 to 100 ms) and an earlier baseline period (−150 to −50 ms). But again, there was neither a significant difference between flanker errors and correct trials (*M =* −0.46 μV, SE = 0.43 μV), *t*(20) = 1.06, *p* = 0.303, *d* = 0.231, nor between nonflanker guesses and correct trials (*M =* −1.09 μV, SE = 0.68 μV), *t*(20) = 1.60, *p* = 0.125, *d* = 0.349, or between flanker errors and nonflanker guesses (*M =* −0.68 μV, SE = 0.70 μV), *t*(20) = 0.96, *p* = 0.348, *d* = 0.209.

Next, we considered the Pe in the waveforms from the centroparietal electrode cluster (Figure 3B,D). There was a clear positivity in flanker errors and nonflanker guesses relative to the correct baseline in the time range of the Pe.^1^ Moreover, this Pe is larger for flanker errors than for nonflanker guesses. Figure 3F shows a clear posterior distribution resembling that in the visible‐target condition. Corroborating these observations, the statistical analyses revealed a significant positivity for flanker errors (*M =* 2.43 μV, SE = 0.40 μV); *t*(20) = 4.29, *p* < 0.001, *d* = 0.936, as well as for nonflanker guesses (*M =* 1.54 μV, SE = 0.3 μV); *t*(20) = 3.68, *p* < 0.001, *d* = 0.803, relative to the correct baseline. Furthermore, this Pe was larger for flanker errors than for nonflanker guesses; *t*(20) = 2.40, *p* = 0.026, *d* = 0.524.

#### 3.2.3 Visible‐Target Condition Versus Invisible‐Target Condition

Finally, we compared Ne/ERN and Pe amplitudes across all conditions in a combined analysis. Only flanker errors were included as only these error types could occur in all conditions. Ne/ERN amplitudes varied significantly across the three SMI conditions, *F*(2, 40) = 10.7, *p* < 0.001, *η*_*p*_^2^ = 0.349, reflecting that the invisible‐target condition (*M =* −0.33 μV, SE = 0.21 μV) showed smaller Ne/ERNs than the 250‐SMI (*M =* −1.88 μV, SE = 0.29 μV), *t*(20) = 4.26, *p* < 0.001, *d* = 0.930, and the 133‐SMI condition (*M =* −0.69 μV, SE = 0.24 μV), *t*(20) = 1.28, *p* = 0.214, *d* = 0.279. In contrast, Pe amplitudes did not vary significantly across conditions, *F*(2, 40) = 0.01, *p* = 0.988, *η*_*p*_^2^ < 0.001. When including both components in a two‐way ANOVA with component (Ne/ERN, Pe) as an additional variable, we obtained significant main effects of SMI condition, *F*(2, 40) = 5.49, *p* = 0.008, *η*_*p*_^2^ = 0.215, but not for component, *F*(1, 20) = 2.35, *p* = 0.141, *η*_*p*_^2^ = 0.105. The interaction between SMI condition and component only reached marginal significance, *F*(2, 40) = 3.14, *p* = 0.054, *η*_*p*_^2^ = 0.136.

## 4 Discussion

Although the Ne/ERN, an early error signal, has been widely studied over the last decades, it is far from clear whether and how the Ne/ERN relates to error awareness. Whereas Di Gregorio et al. (2018) showed that an Ne/ERN is not necessary for the emergence of a Pe, it is still unclear whether this holds for error awareness as well. The main objective of the present study was thus to investigate whether errors can be consciously detected in the absence of an Ne/ERN. To test this, we added an error signaling task to the target‐masking paradigm of Di Gregorio et al. (2018). This paradigm allows for the creation of errors that are detectable without knowing the correct response. As the Ne/ERN is assumed to rely on the representation of a correct response, these error trials should therefore lead to error awareness but not to an Ne/ERN.

Confirming this prediction, our study was able to show that error signaling (and thus error awareness) can occur without an Ne/ERN. Most participants (the good detectors group) detected flanker errors in the invisible‐target condition very reliably, and these detected flanker errors showed no Ne/ERN. This is plausible as the Ne/ERN requires a representation of the correct response, and participants could not know what the correct response was in this condition. This demonstrates that an Ne/ERN is not a necessary precondition for error awareness to emerge. Moreover, these errors were associated with a Pe, which replicates the core result of Di Gregorio et al. (2018) and again confirms that Ne/ERN and Pe are dissociable. This again demonstrates that the two components do not represent stages of a unitary process (e.g., Yeung et al. 2004; Ullsperger, Fischer, et al. 2014) but can be viewed as two independent mechanisms of performance monitoring (Charles et al. 2013; Maier et al. 2015).

Our results can be interpreted within the framework of the evidence accumulation account of error awareness (Desender et al. 2021; Steinhauser and Yeung 2010; Ullsperger et al. 2010), which states that error awareness emerges when the accumulated evidence for an error exceeds a criterion, and that the Pe reflects this accumulated evidence. Whereas the early error signal reflected by the Ne/ERN could form one source of evidence, it is probably not the only one and—as shown by the present data—also not a necessary source of evidence. It is rather conceivable that there are various internal and external sources of evidence such as sensory, proprioceptive, and interoceptive signals that contribute to error awareness (Wessel et al. 2012; Wessel et al. 2011; Ullsperger et al. 2010). In the present paradigm, error detection is an inference‐based process that relies on the explicit knowledge that a response to the flankers must be an error. Other sources of evidence can be interoceptive signals including error‐related changes in heart rate, pupil response, and skin conductance, for which it has already been shown that they can correlate with error awareness (Di Gregorio et al. 2024; Hajcak et al. 2003; Harsay et al. 2018; Quirins et al. 2018; Wessel et al. 2011; but see Maier et al. 2019). Despite the absence of the Ne/ERN, these interoceptive signals could have contributed to error awareness in our study, for instance, by mediating between inference and error awareness.

As in our previous study, we also obtained a Pe for nonflanker guesses; but this Pe was smaller than that for flanker errors in the invisible‐target condition. This can be interpreted by assuming that the Pe amplitude scales with the conditional probability that a response is an error, which is 100% for flanker errors and 50% for nonflanker guesses in the invisible‐target condition (Di Gregorio et al. 2018). This relationship might be a consequence of the fact that the accumulated evidence for an error varies with these probabilities across error types. However, there are also alternative ways to account for this result. It is possible that the reduced Pe for nonflanker guesses reflects an averaging artifact indicating that nonflanker guesses are perceived as correct by some participants but as errors by others. Inspection of the single‐subject data did not reveal a bimodal pattern of the differences between flanker errors and nonflanker guesses; but such a pattern is generally difficult to identify in noisy physiological data. Alternatively, it is also possible that, within each participant, we averaged across single nonflanker guesses which were perceived as an error (full Pe) or as a correct response (no Pe). As the Pe difference between flanker errors and nonflanker guesses appears to be an interesting window into the mechanisms underlying the Pe, future studies would be desirable that shed more light on the origin of this effect.

Results from previous studies were inconsistent with regard to the relationship between error awareness and the Ne/ERN. Some found that signaled errors were associated with a larger Ne/ERN (e.g., Scheffers and Coles 2000; Wessel et al. 2011; Woodman 2010), while others did not find this relationship (e.g., Dhar et al. 2011; Endrass et al. 2007; Nieuwenhuis et al. 2001; Shalgi et al. 2009). It has previously been argued that the occasional observation of such a relationship could reflect a correlative rather than a causal link between Ne/ERN and error awareness, and that this correlation is mediated by features of task performance (e.g., Steinhauser and Yeung 2010; Di Gregorio et al. 2016). For instance, in some tasks, aware errors could be associated with higher postresponse conflict than unaware errors, and this leads to a higher Ne/ERN for the former (Di Gregorio et al. 2016). In the invisible‐target condition, we largely deconfounded conflict and error awareness by preventing the correct response from being activated, and thus, preventing postresponse conflict from occurring (although the presence of residual conflict, e.g., between flanker and nonflanker responses cannot be fully excluded). Accordingly, our results are compatible with the idea that the relationship between Ne/ERN and error awareness in previous studies is mediated by conflict or other variables related to the activation of the correct response. However, our results are also compatible with a second explanation for the inconsistent relationship between Ne/ERN and error awareness. While showing that an Ne/ERN is not necessary for error awareness to emerge, we cannot exclude the possibility that the Ne/ERN still contributes to the evidence for an error under some conditions. Therefore, it is possible that whether the Ne/ERN correlates with error awareness in a specific task depends on whether error awareness in this specific task relies on the Ne/ERN or other sources of evidence.

Unlike in our previous study (Di Gregorio et al. 2018), we observed a pronounced SMI‐effect on performance as well as the EEG. In the good detectors group, the shorter SMI of 133 ms led to impaired primary task performance and error detection, but also a reduced Ne/ERN and a smaller amplitude for correct trials in the Ne/ERN time range (a so‐called CRN; Vidal et al. 2000). This might be due to the inclusion of an error signaling procedure, which was not used in Di Gregorio et al. (2018). Grützmann et al. (2014) showed that error signaling leads to an increased Ne/ERN and CRN and argued that this reflects an enhanced attention to one's accuracy. In the present study, this enhanced attention to one's accuracy could have slightly reduced attention to external stimuli, which might have affected performance and the Ne/ERN particularly for the more difficult 133‐SMI condition.

Another unexpected result of this study is the pronounced individual differences in signaling flanker errors in the invisible‐target condition. Whereas 64% of participants reliably reported these flanker errors (the good detectors group), the remaining participants showed little or no error awareness in this condition (the bad detectors group). The fact that more than one third of the participants had to be excluded for the main analyses is a limitation of the present study as it potentially reduces the generalizability of the results. It is therefore crucial to explain the difficulty of detecting flanker errors in the invisible‐target condition in so many participants. One explanation is that bad detectors applied a higher detection criterion. According to Steinhauser and Yeung (2010), a higher detection criterion implies that fewer errors are detected; however, the averaged evidence across all error trials (detected and undetected) and thus their Pe amplitude remains the same. However, our data seem to contradict this. In the Supporting Information, we compared Pe amplitudes in the invisible‐target condition between good and bad detectors groups. The Pe for flanker errors was actually lower in the bad detectors group. This speaks against the idea of a higher detection criterion in the bad detectors group but rather suggests that the low detection rate in this group relies on an impaired accumulation of evidence for an error. Possibly, these participants either failed to notice that a response to the flanker had occurred or ignored the information that these flanker responses are always errors. Instead, error awareness could have relied more on the activation of the correct response, and thus the Ne/ERN, which was absent in the invisible‐target condition. This receives support from the observation that overall detection rates in the visible‐target condition (in which an Ne/ERN was generated) were similar in bad detectors and good detectors. The two groups did not differ in their general ability to detect errors but rather in collecting evidence for an error in the invisible‐target condition. One has to note that the group differences in the present study have to be treated with caution due to the small sample size in the bad detectors group.^2^

The observed individual differences in the ability to utilize information for metacognition appear to be an interesting field for future research. Interindividual abilities in introspection have previously been found to rely on neuroanatomical differences (e.g., Fleming et al. 2010; Sinanaj et al. 2015). In our specific task, these differences might also be related to differences in working memory capacity, which has been demonstrated to impact error monitoring (Miller et al. 2012). In this vein, other studies have shown that both the Ne/ERN and Pe are affected by working memory load induced by dual‐tasking (Klawohn et al. 2016; Moser et al. 2013; Steinhauser and Steinhauser 2021; Watanabe et al. 2024; Weißbecker‐Klaus et al. 2016) although these results do not always follow a consistent pattern (for a discussion, see Schuch et al. 2019). Interestingly, one study suggests that particularly the identification of flanker errors might be difficult under high working memory load (Maier and Steinhauser 2017). As one could argue that our paradigm places high demands on working memory due to the use of a three‐choice task and the need to signal errors, future studies could measure working memory capacity to account for inter‐individual differences in this paradigm and to investigate whether participants with low working memory capacity rely more on the Ne/ERN rather than task rules to signal errors in our task.

## 5 Conclusion

Taken together, the present study demonstrated that the ability to become aware of an error does not rely on early error processing as reflected by the Ne/ERN, but rather relates to late error processing represented by the Pe. This does not exclude the possibility that error awareness can be influenced by the Ne/ERN but shows that an Ne/ERN is not necessary for error awareness to emerge. These results support the notion that the Ne/ERN and Pe constitute independent systems of error monitoring.

## Author Contributions

**Julia Dumsky:** conceptualization, writing – original draft, formal analysis, visualization, investigation. **Martin E. Maier:** writing – review and editing, conceptualization. **Francesco Di Gregorio:** writing – review and editing, conceptualization. **Marco Steinhauser:** conceptualization, writing – review and editing, supervision, funding acquisition.

## Conflicts of Interest

The authors declare no conflicts of interest.

## Supporting information


**Data S1:** psyp70128‐sup‐0001‐Supinfo01.docx.

**Figure S1:** Distribution of detection frequencies of flanker errors in the invisible‐target condition. Participants with detection frequencies of 40% or above were included in the group of good detectors.

**Figure S2:** Error‐related negativity (Ne/ERN; frontocentral cluster in left column) and error positivity (Pe; centroparietal cluster in right column) in the visible‐target (133/250‐SMI) conditions of the good detectors group. All trials were considered irrespective of whether they were correctly classified or not. (A, B) Waveforms from all response types. (C, D) Difference waves for flanker errors (minus corrects) and nonflanker errors (minus corrects). (E, F) Topographies of the difference waves in the time range of the Ne/ERN and Pe. Gray areas indicated the time range of the Ne/ERN and Pe in each graph. CP = centroparietal, CR = correct response, FC = frontocentral, FE = flanker error, NFE = nonflanker error, SMI = stimulus‐mask interval.

**Figure S3:** Error‐related negativity (Ne/ERN; frontocentral cluster in left column) and error positivity (Pe; centroparietal cluster in right column) in the visible‐target (133/250‐SMI) conditions of the bad detectors group. All trials were considered irrespective of whether they were correctly classified or not. (A, B) Waveforms from all response types. (C, D) Difference waves for flanker errors (minus corrects) and nonflanker errors (minus corrects). (E, F) Topographies of the difference waves in the time range of the Ne/ERN and Pe. Gray areas indicated the time range of the Ne/ERN and Pe in each graph. CP = centroparietal, CR = correct response, FC = frontocentral, FE = flanker error, NFE = nonflanker error, SMI = stimulus‐mask interval.

**Figure S4:** Error‐related negativity (Ne/ERN; frontocentral cluster in left column) and error positivity (Pe; centroparietal cluster in right column) in the invisible‐target (0‐SMI) condition of the good detectors group. All trials were considered irrespective of whether they were correctly classified or not. Data for correct responses were taken from the 133‐SMI condition. (A, B) Waveforms from all response types. (C, D) Difference waves for flanker errors (minus corrects) and nonflanker guesses (minus corrects). (E, F) Topographies of the difference waves in the time range of the Ne/ERN and Pe. Gray areas indicated the time range of the Ne/ERN and Pe in each graph. CP = centroparietal, CR = correct response, FC = frontocentral, FE = flanker error, NFG = nonflanker guess, SMI = stimulus‐mask interval.

**Figure S5:** Error‐related negativity (Ne/ERN; frontocentral cluster in left column) and error positivity (Pe; centroparietal cluster in right column) in the invisible‐target (0‐SMI) condition of the bad detectors group. All trials were considered irrespective of whether they were correctly classified or not. Data for correct responses were taken from the 133‐SMI condition. (A, B) Waveforms from all response types. (C, D) Difference waves for flanker errors (minus corrects) and nonflanker guesses (minus corrects). (E, F) Topographies of the difference waves in the time range of the Ne/ERN and Pe. Gray areas indicated the time range of the Ne/ERN and Pe in each graph. CP = centroparietal, CR = correct response, FC = frontocentral, FE = flanker error, NFG = nonflanker guess, SMI = stimulus‐mask interval.

**Table S1:** Primary task Performance.

**Table S2:** Frequencies of detection types and trial numbers for flanker errors and nonflanker errors in the visible‐target condition for both subgroups.

**Table S3:** Frequencies of detection types and trial numbers for flanker errors and nonflanker guesses in the invisible‐target condition for both subgroups.

## Data Availability

The data that support the findings of this study are available from the corresponding author upon reasonable request.

## Endnotes

^1^
Figure 3 shows that the centroparietal cluster (Figs. 4BD) is associated with more noise than the frontocentral cluster (Figs. 4 AC). A closer inspection of the data revealed that this is due to an enhanced noise level at electrode P2 in five participants, which however was not strong enough to meet our interpolation criterion. Importantly, re‐analysis of the data without this electrode did not change the results.

^2^
If bad detectors do not take into account whether a response to the flanker has occurred, they should assign an error probability of 66% to either of the three responses (irrespective of whether it is a flanker error or nonflanker guess). As this is higher than the 50% error probability assigned to nonflanker guesses by good detectors, one would expect that the Pe on nonflanker guesses is higher for good detectors than for bad detectors. This was not observed which might reflect the restricted power to find such a small effect given the small sample sizes or to the fact that error awareness in bad detectors relied more on early error processing as discussed.
