# Supplementary material for: Error Awareness Can Occur in the Absence of an Error‐Related Negativity
Source: Psychophysiology. 2025 Oct 7;62(10):e70128. doi: 10.1111/psyp.70128 (PMC12504923; doi:10.1111/psyp.70128)
Supplement: Supplementary file 1 — Data S1: psyp70128‐sup‐0001‐Supinfo01.docx. [file PSYP-62-e70128-s004.docx]

# Supplementary Material

To investigate whether error awareness can occur without an Ne/ERN, we analyzed detected flanker errors in the invisible-target condition. However, this was possible only in 21 of the 33 participants as only these participants (good detectors group) had a sufficient detection rate for these errors. The remaining 12 participants (bad detectors group) could not be analyzed in this way as the number of detected flanker errors in the invisible-target condition was too low. In the following, we compare the two groups with respect to behavioral and EEG data and describe how the two groups were formed.

## Behavioral data

The results of the primary task performance are presented in Table S1, and the detection rates in the visible-target and invisible-target conditions are shown in Table S2 and S3, respectively. The latter tables include mean trial numbers for each condition. For these behavioral data, we repeated all analyses with the additional variable group (good detectors, bad detectors). Only significant effects involving this additional variable are reported. If an interaction with group was significant, groups were analyzed separately. The analyses are reported in the same order as in the main text. The criteria for creating the two groups are discussed when describing the results from the invisible-target condition.

**Visible-target condition (133/250-SMI).** No significant effects involving the variable group were obtained for RTs, error rates, or the proportion of flanker errors in the visible-target condition. However, group differences emerged for the rates of error detection. Submitting the rate of detected errors to a three-way mixed ANOVA with the variables error type, SMI and group revealed a significant interaction between SMI and group, *F*(1, 31) = 5.05, *p* = .032, *η_p_*^2^ = .140. The rate of detected errors was similar across SMIs for bad detectors, *t*(11) = 0.37, *p* = .717, *d* = 0.108, but was significantly lower for the 133-SMI condition (*M* = 81.1%, *SE* = 2.4%) than for the 250-SMI condition (*M* = 89.0%, *SE* = 2.2%) for the good detectors, *t*(20) = 3.81, *p* = .001, *d* = 0.831. A different pattern was obtained when considering the rate of errors classified as “unsure”. The same ANOVA as above again revealed a significant two-way interaction between SMI and group, *F*(1, 31) = 5.28, *p* = .029, *η_p_*^2^ = .146. The rate of errors classified as “unsure” was similar across SMIs for bad detectors, *t*(11) = 0.56, *p* = .586, *d* =0 .092, but was significantly higher for the 133-SMI condition (*M* = 11.2%, *SE* = 1.5%) than for the 250-SMI condition (*M* = 6.3%, *SE* = 1.3%) for good detectors, *t*(20) = 2.90, *p* = .009, *d* =0 .633. No significant effects involving group were obtained for correct trials that were classified as “error” or correct trials that were classified as “unsure”. Taken together, the SMI had a detrimental effect on error signaling in the good detectors group but not in the bad detectors group.

**Invisible-target condition (0-SMI).** The rate of flanker errors was higher for bad detectors (*M* = 38.9%, *SE* = 3.3%) than for good detectors (*M* = 31.4%, *SE* = 1.4%), *t*(31) = 2.48, *p* = .019, *d* = 0.438. Moreover, subjecting RTs to a two-way mixed ANOVA with the variables error type and group revealed a significant interaction, *F*(1, 31) = 8.06, *p* = .008, *η_p_*^2^ = .206. Whereas similar RTs were obtained for flanker errors and nonflanker guesses in the bad detectors group, *t*(12) = 1.02, *p* = .330, *d* = 0.283, flanker errors showed lower RTs than nonflanker guesses in the good detectors group, *t*(20) = 5.73, *p* < .001, *d* = 1.250.

Before we compare the two groups with respect to secondary task performance in the invisible-target condition, we discuss how the two groups were formed. Detected flanker errors in the invisible-target condition are a crucial condition for the present study, but a closer look at the data indicates that the detection rate for this condition differs strongly across participants. Figure S1 reveals a bimodal distribution of these detection rates across participants. Whereas most participants detected flanker errors with a medium to high frequency, others almost completely failed to detect these errors. Because we were ultimately interested in error-related brain activity in detected errors, which were sufficiently available only in the former participants, we had to split our sample into the two mentioned subgroups. The 21 participants with a detection rate of more than 40% were assigned to the good detectors group whereas the remaining 12 participants were included in the bad detectors group. Table S3 shows that only good detectors have sufficient trial numbers for detected flanker errors (72.8 trials on average; range: 38 – 111 trials) to conduct analyses of Ne/ERN and Pe amplitudes in the invisible target condition, while bad detectors lack the necessary trial numbers in this condition (4.8 trials on average).

Table S3 characterizes the two groups in terms of secondary task performance and trial numbers. A two-way mixed ANOVA on the detection rates (i.e., proportion of trials classified as “errors”) with group (good detectors, bad detectors) and trial type (flanker error, nonflanker guesses) revealed a significant interaction, *F*(1, 31) = 129.4, *p* < .001, *η_p_*^2^ = .807. While the detection rate was higher for flanker errors than for non-flanker guesses in good detectors, *t*(20) = 18.6, *p* < .001, *d* = 4.059, it was comparably low in both trial types for bad detectors, *t*(11) = 0.174, *p* = .998, *d* = 0.050. The same ANOVA applied to the rate of trials classified as “unsure” also showed a significant interaction, *F*(1, 31) = 90.0, *p* < .001, *η_p_*^2^ = .744. The rate was higher for nonflanker guesses than for flanker errors in good detectors, *t*(20) = 15.8, *p* < .001, *d* = 3.448, but was comparably high for both trial types in bad detectors, *t*(11) = 0.07, *p* = .999, *d* = 0.020. Finally, for the rate of trials classified as “correct”, no significant main effect or interaction involving group was observed.

## ERP data

For the group analysis of ERP data, we included all trials irrespective of whether trials were correctly classified by the participants or not. Because this implies that the results could differ from the original analyses in the main text, we first conducted each analysis without the variable group and report significant effects. We then added the variable group and report whether significant effects involving this variable were obtained. In this case, separate analyses for each group are provided.

Visible-target conditions (133/250 SMI). Waveforms for all response types (correct, flanker error, nonflanker error) from the visible-target condition together with difference waves and topographies are provided in Figure S2 for the good detectors group and in Figure S3 for the bad detectors group. The two-way ANOVA on the difference between errors and correct trials in the time range of the Ne/ERN revealed a significant effect of SMI, *F*(1*,*32)= 7.70, *p* = .009, *η_p_*^2^ = .194, with a larger Ne/ERN for the 250-SMI condition (*M* = -1.52 μV, *SE* = 0.96 μV) than for the 133-SMI condition (*M* = -0.71 μV, *SE* = 0.63 μV), but no main effect of error type, *F*(1*,*32)= 0.04, *p* = .844, *η_p_*^2^ = .001, and no interaction, *F*(1*,*32)= 0.02, *p* = .894, *η_p_*^2^ < .001. Again, when only waveforms for correct trials were considered, a larger amplitude was found for the 250-SMI condition (*M* = 1.03 μV, *SE* = 0.32 μV) than for the 133-SMI condition (*M* = 0.51 μV, *SE* = 0.28 μV), *t*(32) = 2.64, *p* = .013, *d* = 0.460. With the additional variable group (good detectors, bad detectors), no significant interactions with group were found in any of these analyses.

The two-way ANOVA on the difference between errors and correct trials in the time range of the Pe now revealed a significant effect of SMI, *F*(1*,*32) = 5.84, *p* = .022, *η_p_*^2^ = .154, with a larger Pe for the 250-SMI condition (*M* = 3.51 μV, *SE* = 0.31 μV) than for the 133-SMI condition (*M* = 2.35 μV, *SE* = 0.30 μV). No main effect of error type, *F*(1*,*32) = 1.09, *p* = .304, *η_p_*^2^ = .033, and no interaction was obtained, *F*(1*,*32) = 2.28, *p* = .141, *η_p_*^2^ = .067. When including group as additional variable in this analysis, the two-way interaction between SMI and group became significant, *F*(1*,*31) = 5.03, *p* = .032, *η_p_*^2^ = .140. We therefore conducted this two-way ANOVA separately for each group. As in the main analysis, the good detectors showed no significant effects of SMI, *F*(1, 20) = 0.08, *p* = .774, *η_p_*^2^ = .004, or error type, *F*(1, 20) = 0.60, *p* = .446, *η_p_*^2^ = .029, and no significant interaction, *F*(1, 20) = 0.42, *p* = .522, *η_p_*^2^ = .021 (Fig. S2BD). Please note that the exact results slightly differ from the main analysis, because here, we included both correctly and incorrectly classified trials, whereas in the main analysis, we included only correctly classified trials. The bad detectors showed no significant effect of error type, *F*(1, 11) = 0.29, *p* = .600, *η_p_*^2^ = .026, but a significant effect of SMI, *F*(1, 11) = 4.99, *p* = .047, *η_p_*^2^ = .312, with a higher Pe for the 250-SMI condition than for the 133-SMI condition (Fig. S3BD). The interaction was not significant, *F*(1, 11) = 2.11, *p* = .174, *η_p_*^2^ = .161. Again, we analyzed only correct waveforms in the time range of the Pe, which revealed a significantly larger amplitude for the 133-SMI condition (*M* = -2.79 μV, *SE* = 0.35 μV) than for the 250-SMI condition (*M* = -3.65 μV, *SE* = 0.41 μV), *t*(32) = 3.44, *p* = .002, *d* = 0.599. Analyzing correct waveforms in a two-way ANOVA with the variables SMI and group revealed a significant interaction, *F*(1*,*31) = 5.58, *p* = .025, *η_p_*^2^ < .153, indicating a significant effect of SMI for the bad detector group (Fig. S3B), *t*(11) = 3.18, *p* = .009, *d* = 0.918, but not for the good detector group (Fig. S2B), *t*(20) = 0.77, *p* = .452, *d* = 0.168.

Taken together, in the visible-target condition the two groups showed similar effects in the time range of the Ne/ERN but differed with respect to the Pe. Regarding the latter, while no effects of SMI were obtained in the good detectors group, the bad detectors group showed a higher Pe in the 250-SMI condition which was mainly due to a higher amplitude on correct waveforms in the 133-SMI condition.

**Invisible-target condition (0 SMI).** As in the main analysis, we used the correct trials of the 133-SMI condition as baseline for analyzing the invisible-target condition. Waveforms for all respective response types (correct, flanker errors, nonflanker guesses) together with difference waves and topographies are provided in Figure S4 for the good detectors group and in Figure S5 for the bad detectors group. In the time range of the Ne/ERN, neither the difference between flanker errors and correct trials (*M = -*0.01 μV, *SE* = 0.18 μV) reached significance, *t*(32) = 0.06, *p* = .949, *d* = 0.010, nor the difference between nonflanker guesses and correct trials (*M = -*0.2 μV, *SE* = 0.16 μV), *t*(32) = 0.36, *p* = .722, *d* = 0.063. There was also no significant difference between flanker errors and non-flanker guesses, *t*(32) = 0.89, *p* = .378, *d* = 0.155. The variable group was included by subjecting the difference wave between each response type and the respective correct baseline to a two-way ANOVA with the variables response type and group. The interaction between both variables did not reach significance, *F*(1*,* 31) = 3.82, *p* = .060, *η_p_*^2^ = .110.

In the time range of the Pe, the analyses revealed significant differences between flanker errors and corrects (*M =* 1.71 μV, *SE* = 0.38 μV), *t*(32) = 4.55, *p* < .001, *d* = 0.792, as well as between nonflanker guesses and corrects (*M =* 1.31 μV, *SE* = 0.30 μV), *t*(32) = 4.38, *p* < .001, *d* = 0.762. The difference between flanker errors and nonflanker guesses was not significant, *t*(32) = 1.77, *p* = .086, *d* = 0.308. However, the two-way ANOVA showed an interaction between response type and group, *F*(1*,*31) = 7.04, *p* = .013, *η_p_*^2^ = 0.185. The Pe was higher for flanker errors than for nonflanker guesses in the good detectors group (Fig. S4BD), *t*(20) = 2.87, *p* = .009, *d* = 0.626, but not in the bad detectors group (Fig. S5BD), *t*(11) = 1.58, *p* = .142, *d* = 0.456. Moreover, the Pe for flanker errors was higher in the good-detectors group than in the bad detectors group, *t*(31) = 2.33, *p* = .027, *d* = 0.412, whereas the Pe for nonflanker guesses was similar in both groups, *t*(31) = 0.99, *p* = .331, *d* = 0.175.

To summarize, as the visible-target condition, the invisible-target condition shows no group differences in the time range of the Ne/ERN. Regarding the Pe, however, the bad detectors group did not show the pattern of an increased Pe for flanker errors relative to nonflanker guesses that we obtained for the good detectors group.

**Visible-target condition vs. invisible-target condition.** In a final step, we compared flanker errors across all three SMI conditions as in the main analysis. For the Ne/ERN, the one-way ANOVA with the variable SMI (250-SMI, 133-SMI, 0-SMI) revealed a significant effect, *F*(2*,* 64) = 11.5, *p* < .001, *η_p_*^2^ = .264, indicating a lower Ne/ERN in the 0-SMI condition (*M* = -0.01 μV, *SE* = 0.22 μV) than in the 250-SMI condition (*M* = -1.52 μV, *SE* = 0.27 μV), *t*(32) = 4.06, *p* < .001, *d* = 0.707, and in the 133-SMI condition (*M* = -0.73 μV, *SE* = 0.17 μV), *t*(32) = 2.44, *p* = .020, *d* = 0.425. In this analysis, the 250-SMI condition also showed a higher Ne/ERN than the 133-SMI condition, *t*(32) = 2.44, *p* = .020, *d* = 0.425. When adding the variable group, no significant interaction was obtained, *F*(2, 62) = 1.03, *p* = .362, *η_p_*^2^ = .032. For the Pe, we now obtained a significant main effect of SMI, *F*(2*,* 62) = 5.76, *p* = .005, *η_p_*^2^ = .157, and SMI interacted significantly with group when the latter variable was added, *F*(2, 62) = 9.50, *p* < .001, *η_p_*^2^ = .235. Separate analyses for each group revealed that the Pe did not differ significantly across SMIs for the good detectors group, *F*(2, 40) = 0.01, *p* = .992, *η_p_*^2^ < .001, but for the bad detectors group, *F*(2, 22) = 11.7, *p* < .001, *η_p_*^2^ = .515. In the latter, the Pe was smaller for the 0-SMI condition than for the 250-SMI condition, *t*(11) = 4.00, *p* = .002, *d* = 1.152, and the 133-SMI condition, *t*(11) = 5.85, *p* < .001, *d* = 1.689.

Next, we included the Ne/ERN and Pe for flanker errors from each SMI-condition in a two-way ANOVA with the variables SMI and component (Ne/ERN, Pe). We obtained a significant main effect for component, *F*(1, 32) = 71.8, *p* < .001, *η_p_*^2^ = .692, but not for SMI, *F*(2, 64) = 0.20, *p* = .817, *η_p_*^2^ = .006. Most important, the interaction between SMI and component reached significance, *F*(2, 64) = 12.7, *p* < .001, *η_p_*^2^ = .284, which shows that the Ne/ERN and Pe varied differently across conditions. When additionally adding the variable group, the corresponding three-way ANOVA resulted in a significant two-way interaction between SMI and group, *F*(2, 62) = 8.04, *p* < .001, *η_p_*^2^ = .206, and a significant three-way interaction, *F*(2, 62) = 5.96, *p* = .004, *η_p_*^2^ = .161. Separate analyses of each group revealed interactions between SMI and component for the good detectors group, *F*(2, 40) = 4.02, *p* = .026, *η_p_*^2^ = .167, as well as the bad detectors group, *F*(2, 22) = 11.1, *p* < .001, *η_p_*^2^ = .502.

To summarize, the analyses of all trials and participants replicated the dissociation of the Ne/ERN and Pe that was found in Di Gregorio et al. (2018) and in the analyses of correctly classified trials in the good detectors group in the main text. However, we also found that the bad detectors group differed in some respects from the good detectors group. Most notably, the bad detectors group did not show the pattern of Pe amplitudes across response types that characterized the good detectors group. In good detectors, the Pe was larger for flanker errors than for nonflanker guesses mirroring that the probability of an error was 100% in the former but only 50% in the latter. In contrast, bad detectors did not show this difference. The Pe for flanker errors was similar as that for nonflanker guesses and smaller than the Pe in the visible-target condition.

Figure S1. Distribution of detection frequencies of flanker errors in the invisible-target condition. Participants with detection frequencies of 40% or above were included in the group of good detectors.
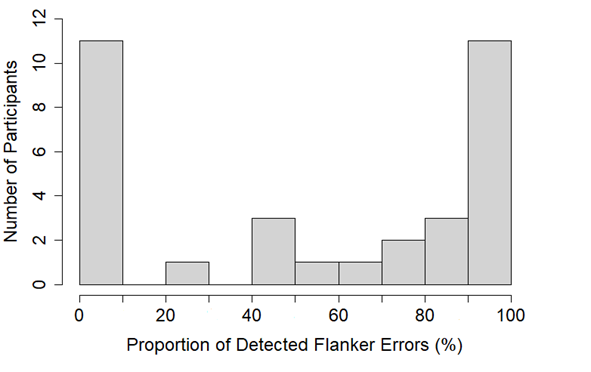


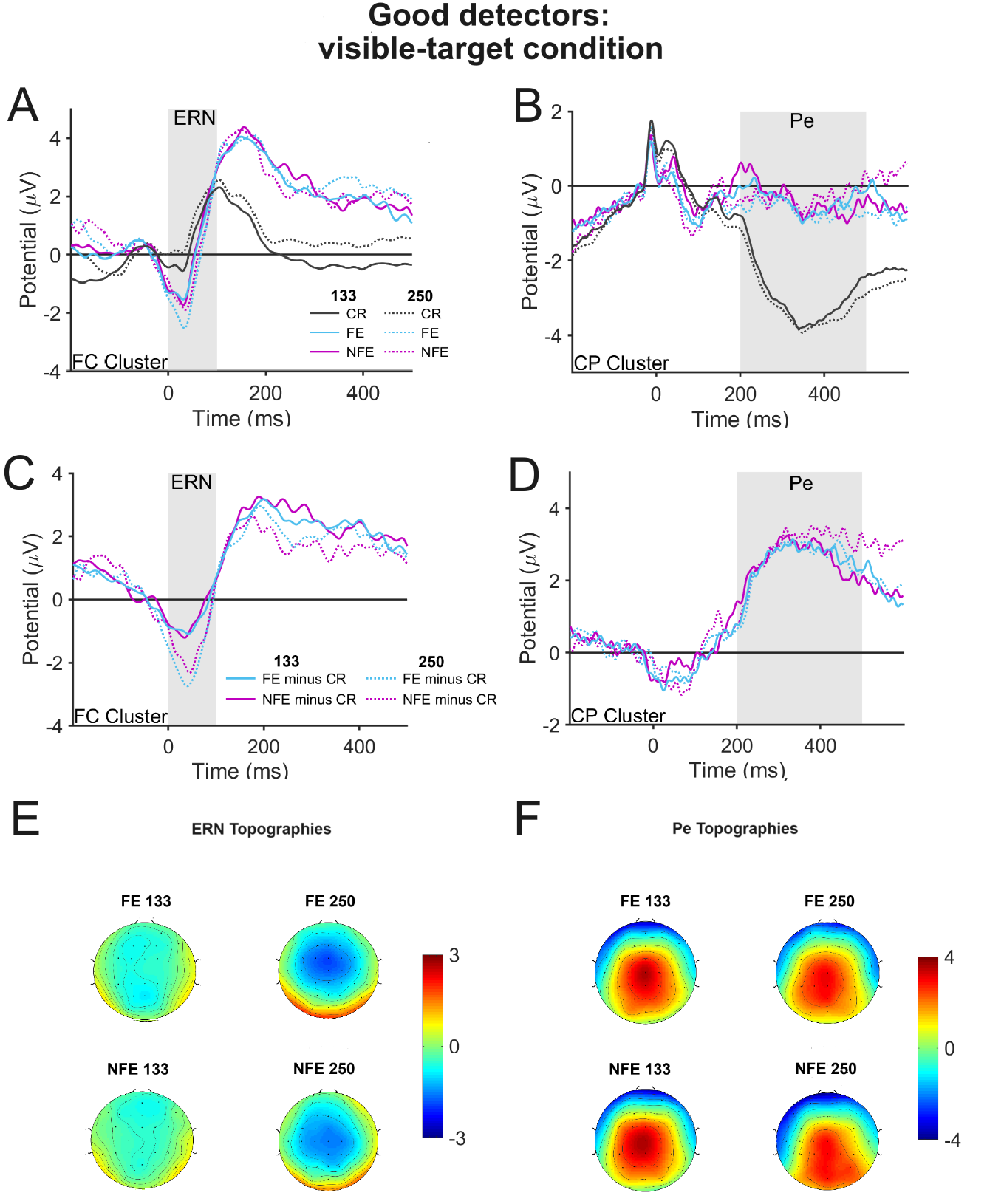


Figure S2. Error-related negativity (Ne/ERN; frontocentral cluster in left column) and error positivity (Pe; centroparietal cluster in right column) in the visible-target (133/250-SMI) conditions of the good detectors group. All trials were considered irrespective of whether they were correctly classified or not. AB: Waveforms from all response types. CD: Difference waves for flanker errors (minus corrects) and nonflanker errors (minus corrects). EF: Topographies of the difference waves in the time range of the Ne/ERN and Pe. Grey areas indicated the time range of the Ne/ERN and Pe in each graph. SMI = stimulus-mask interval, CR = correct response, FE = flanker error, NFE = nonflanker error, FC = frontocentral, CP = centroparietal.


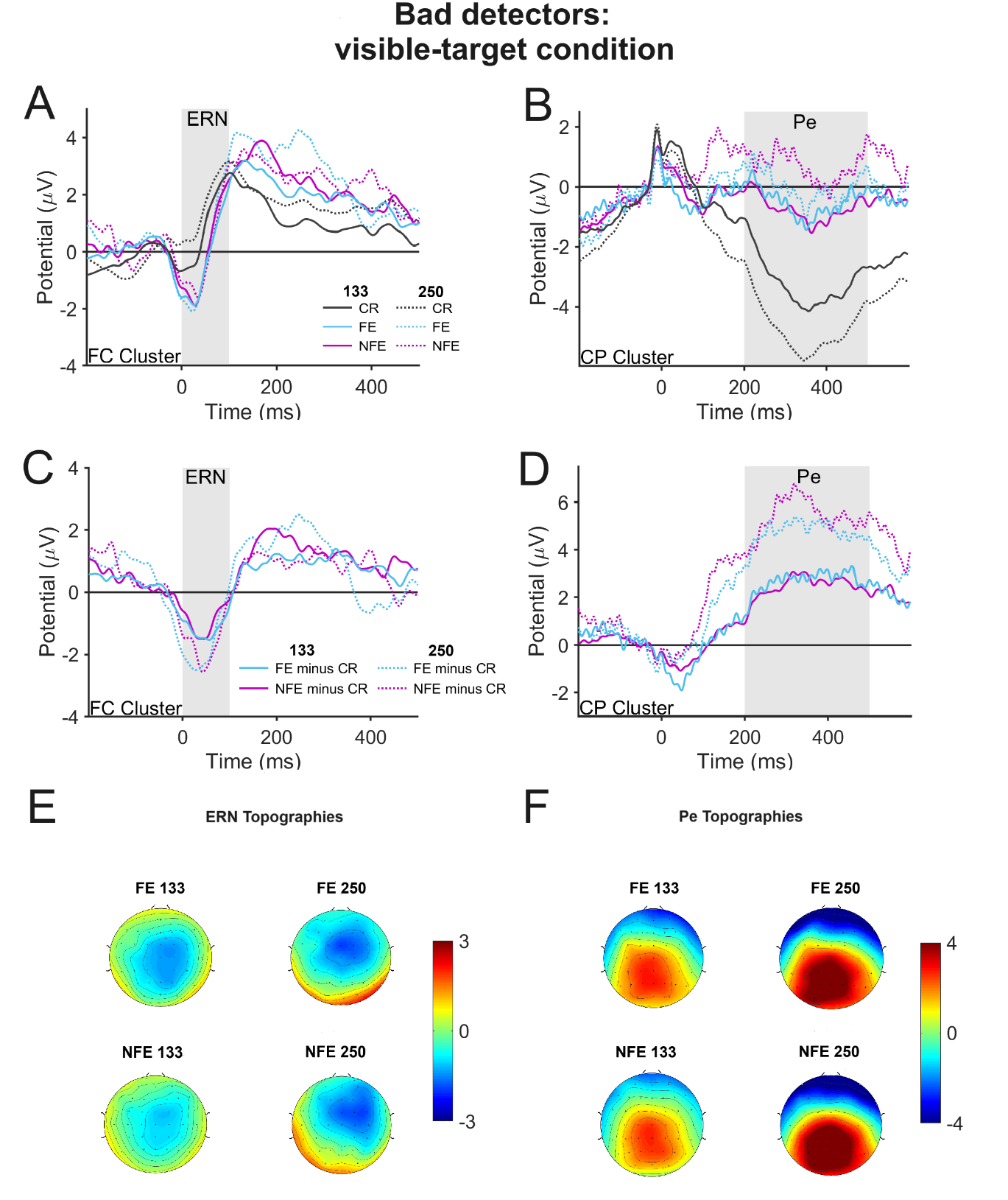


Figure S3. Error-related negativity (Ne/ERN; frontocentral cluster in left column) and error positivity (Pe; centroparietal cluster in right column) in the visible-target (133/250-SMI) conditions of the bad detectors group. All trials were considered irrespective of whether they were correctly classified or not. AB: Waveforms from all response types. CD: Difference waves for flanker errors (minus corrects) and nonflanker errors (minus corrects). EF: Topographies of the difference waves in the time range of the Ne/ERN and Pe. Grey areas indicated the time range of the Ne/ERN and Pe in each graph. SMI = stimulus-mask interval, CR = correct response, FE = flanker error, NFE = nonflanker error, FC = frontocentral, CP = centroparietal.


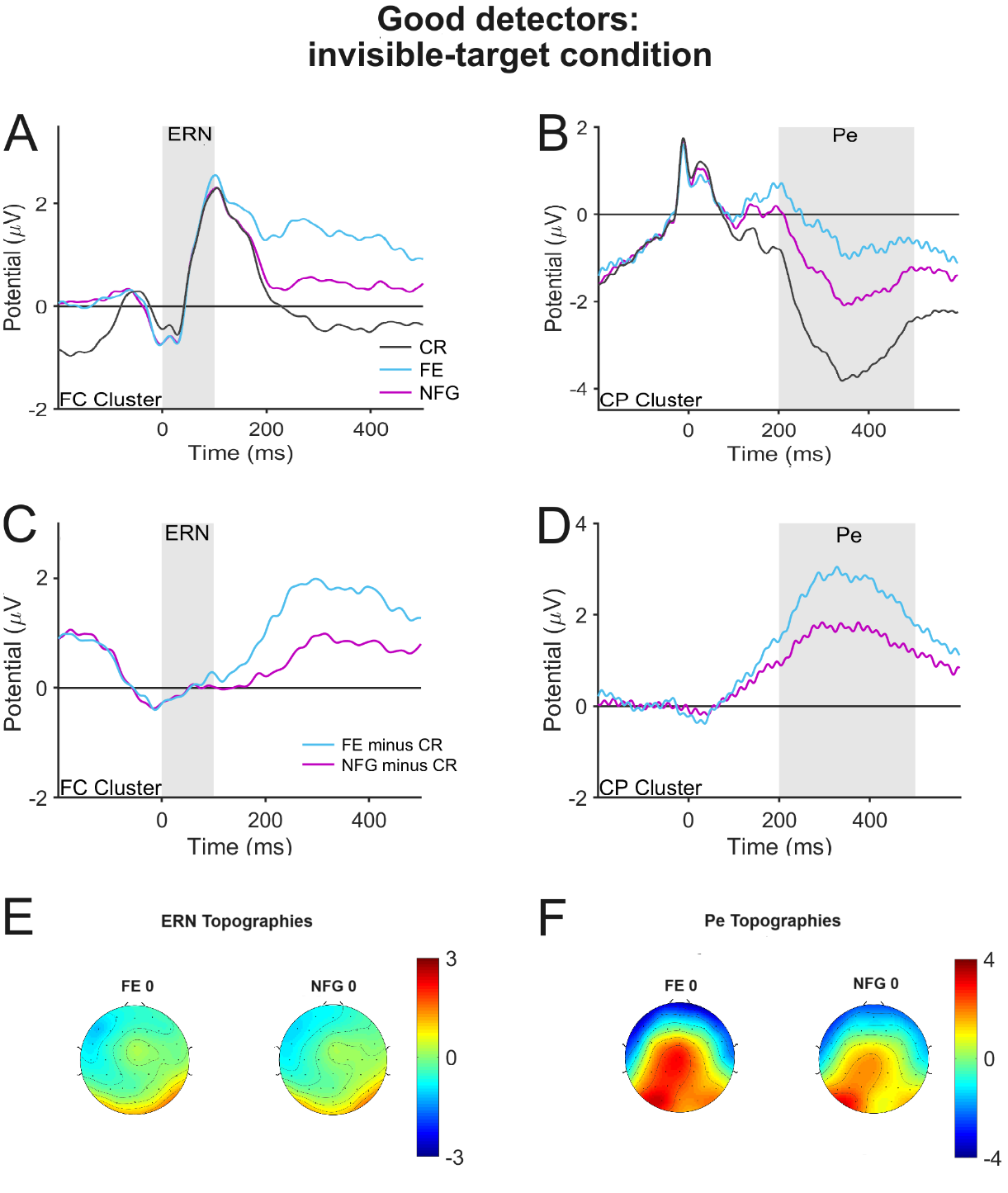


Figure S4. Error-related negativity (Ne/ERN; frontocentral cluster in left column) and error positivity (Pe; centroparietal cluster in right column) in the invisible-target (0-SMI) condition of the good detectors group. All trials were considered irrespective of whether they were correctly classified or not. Data for correct responses were taken from the 133-SMI condition. AB: Waveforms from all response types. CD: Difference waves for flanker errors (minus corrects) and nonflanker guesses (minus corrects). EF: Topographies of the difference waves in the time range of the Ne/ERN and Pe. Grey areas indicated the time range of the Ne/ERN and Pe in each graph. SMI = stimulus-mask interval, CR = correct response, FE = flanker error, NFG = nonflanker guess, FC = frontocentral, CP = centroparietal.


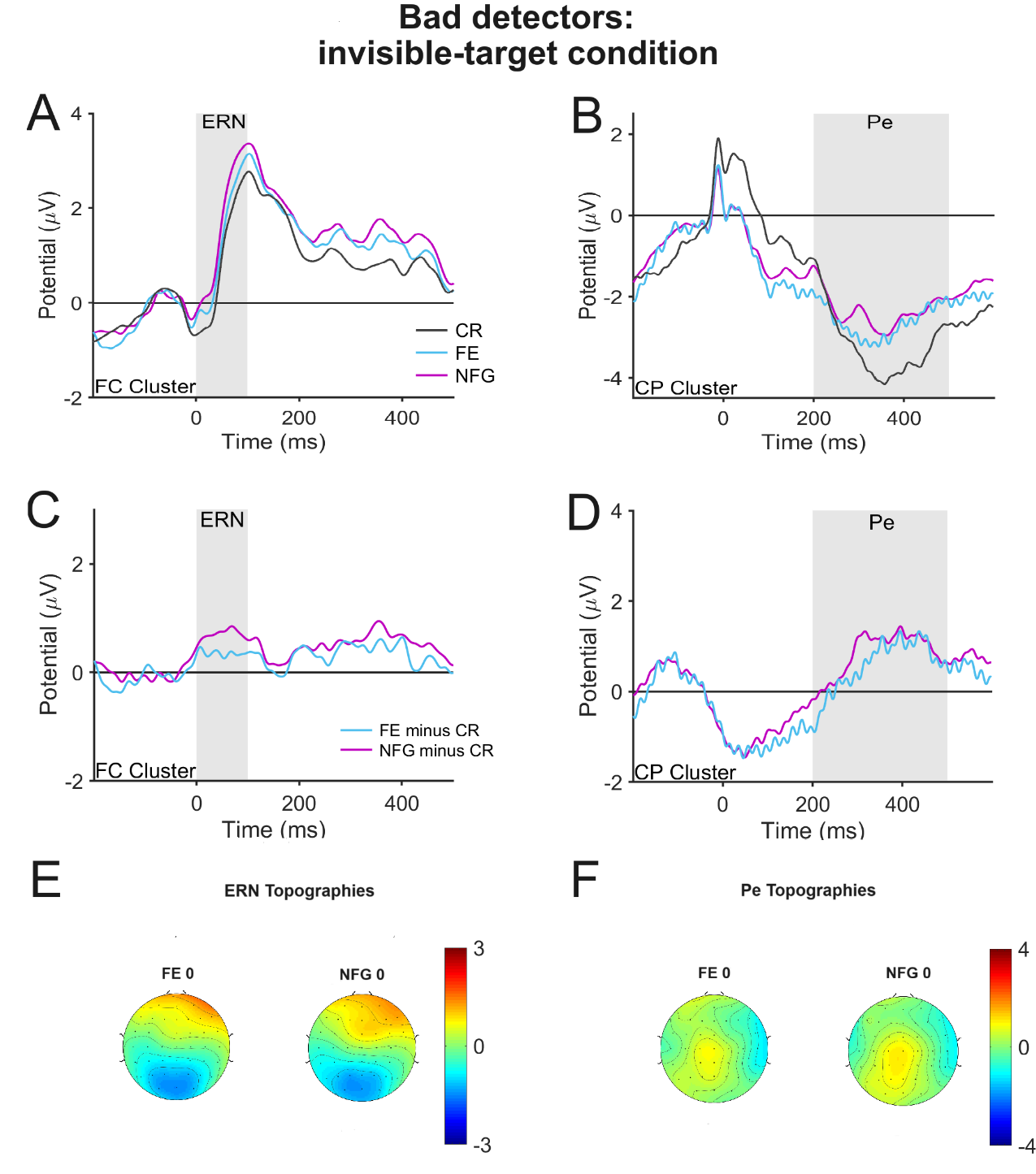


Figure S5. Error-related negativity (Ne/ERN; frontocentral cluster in left column) and error positivity (Pe; centroparietal cluster in right column) in the invisible-target (0-SMI) condition of the bad detectors group. All trials were considered irrespective of whether they were correctly classified or not. Data for correct responses were taken from the 133-SMI condition. AB: Waveforms from all response types. CD: Difference waves for flanker errors (minus corrects) and nonflanker guesses (minus corrects). EF: Topographies of the difference waves in the time range of the Ne/ERN and Pe. Grey areas indicated the time range of the Ne/ERN and Pe in each graph. SMI = stimulus-mask interval, CR = correct response, FE = flanker error, NFG = nonflanker guess, FC = frontocentral, CP = centroparietal.

**Table S1.** Primary Task Performance

| **Conditions** | **Error Rates (%)** | **Prop. FE (%)** | **RT Correct (ms)** | **RT FE**  **(ms)** | **RT NFE/G (ms)** |
| --- | --- | --- | --- | --- | --- |
| **Good Detectors** |  |  |  |  |  |
| 250-SMI | 16.0 (±1.7) | 57.2 (±2.3) | 537 (±17) | 531 (±33) | 523 (±31) |
| 133-SMI | 22.5 (±1.6) | 56.8 (±1.5) | 563 (±18) | 551 (±34) | 537 (±27) |
| 0-SMI | - | 31.4 (±1.4) | - | 551 (±22) | 603 (±28) |
| **Bad**  **Detectors** |  |  |  |  |  |
| 250-SMI | 16.4 (±3.1) | 53.9 (±2.3) | 499 (±15) | 492 (±35) | 501 (±48) |
| 133-SMI | 26.9 (±2.7) | 54.9 (±1.4) | 512 (±17) | 479 (±23) | 490 (±25) |
| 0-SMI | - | 38.9 (±3.3) | - | 445 (±16) | 456 (±25) |

*Note.* NFE/G refers to NFE in the 250-SMI and 133-SMI conditions but NFG in the 0-SMI condition. SMI = stimulus-masking interval, ms = milliseconds, RT = response time, FE = flanker error, NFE = nonflanker error, NFG = nonflanker guess, Prop. = proportion. Within-participants standard errors of the mean are provided in parentheses.

**Table S2**. Frequencies of Detection Types and Trial Numbers for Flanker Errors and Nonflanker Errors in the Visible-Target Condition for Both Subgroups

| **Conditions** | **Frequencies of Detection Types in % and Averaged Trial Numbers** | | |  |
| --- | --- | --- | --- | --- |
|  | **Good Detectors** | **Trials** | **Bad Detectors** | **Trials** |
| **250-SMI** |  |  |  |  |
| **Flanker**  **Errors** |  |  |  |  |
| „correct“ | 4.3 (± 2.1) | 1.2 | 3.2 (± 1.3) | 0.6 |
| „error“ | 88.6 (± 3.1) | 22.2 | 90.8 (± 2.2) | 24.3 |
| „unsure“ | 7.1 (± 1.8) | 1.7 | 6 (± 2.1) | 1.3 |
| **Nonflanker Errors** |  |  |  |  |
| „correct“ | 5.2 (± 2) | 1.3 | 1.5 (± 0.9) | 0.4 |
| „error“ | 89.4 (± 3.2) | 18.4 | 89.8 (± 3.6) | 19 |
| „unsure“ | 5.4 (± 2) | 1.2 | 8.6 (± 3.3) | 1.6 |
|  | **Good Detectors** | **Trials** | **Bad Detectors** | **Trials** |
| **133-SMI** |  |  |  |  |
| **Flanker**  **Errors** |  |  |  |  |
| „correct“ | 10.1 (± 2.8) | 3.4 | 4 (± 1) | 1.8 |
| „error“ | 79.4 (± 3.6) | 29.7 | 89.7 (± 2.5) | 37.7 |
| „unsure“ | 10.5 (± 2) | 4 | 6.2 (± 2) | 2.9 |
| **Nonflanker Errors** |  |  |  |  |
| „correct“ | 5.3 (± 1.3) | 1.5 | 3.4 (± 1.1) | 1.3 |
| „error“ | 82.9 (± 3.2) | 23 | 90.4 (± 2.3) | 31.8 |
| „unsure“ | 11.9 (± 2.3) | 3.4 | 6.2 (± 1.9) | 2.3 |

*Note.* SMI = stimulus-masking interval. Within-participants standard errors of the mean are provided in parentheses.

**Table S3**. Frequencies of Detection Types and Trial Numbers for Flanker Errors and Nonflanker Guesses in the Invisible-Target Condition for Both Subgroups

| **Conditions** | **Frequencies of Detection Types in % and Averaged Trial Numbers** | | |  |
| --- | --- | --- | --- | --- |
|  | **Good Detectors** | **Trials** | **Bad Detectors** | **Trials** |
| **Flanker Errors** |  |  |  |  |
| „correct“ | 0.8 (±0.3) | 0.8 | 11.3 (±7.6) | 20.3 |
| „error“ | 81.6 (±4.3) | 72.8 | 3.9 (±2.3) | 4.8 |
| „unsure“ | 17.7 (±4.2) | 16.8 | 84.9 (±8.4) | 86.8 |
| **Nonflanker Guesses** |  |  |  |  |
| „correct“ | 4.2 (±1.6) | 8.2 | 11.2 (±5.9) | 16.2 |
| „error“ | 2.9 (±0.7) | 5.5 | 5.2 (±3.5) | 5.8 |
| „unsure“ | 93.0 (±1.7) | 184 | 83.6 (±8.2) | 154.2 |

*Note*. Within-participants standard errors of the mean are provided in parentheses.
