# Supplementary material for: Error Awareness Can Occur in the Absence of an Error‐Related Negativity
Source: Psychophysiology. 2025 Oct 7;62(10):e70128. doi: 10.1111/psyp.70128 (PMC12504923; doi:10.1111/psyp.70128)
Supplement: Supplementary file 2 — Figure S1: Distribution of detection frequencies of flanker errors in the invisible‐target condition. Participants with detection frequencies of 40% or above were included in the group of good detectors. [file PSYP-62-e70128-s009.docx]

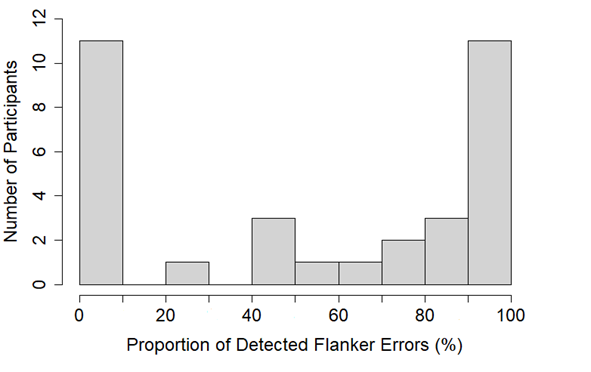


**Figure S1.** Distribution of detection frequencies of flanker errors in the invisible-target condition. Participants with detection frequencies of 40% or above were included in the group of good detectors.
