# Supplementary material for: Error Awareness Can Occur in the Absence of an Error‐Related Negativity
Source: Psychophysiology. 2025 Oct 7;62(10):e70128. doi: 10.1111/psyp.70128 (PMC12504923; doi:10.1111/psyp.70128)
Supplement: Supplementary file 6 — Figure S5: Error‐related negativity (Ne/ERN; frontocentral cluster in left column) and error positivity (Pe; centroparietal cluster in right column) in the invisible‐target (0‐SMI) condition of the bad detectors group. All trials were considered irrespective of whether they were correctly classified or not. Data for correct responses were taken from the 133‐SMI condition. (A, B) Waveforms from all response types. (C, D) Difference waves for flanker errors (minus corrects) and nonflanker guesses (minus corrects). (E, F) Topographies of the difference waves in the time range of the Ne/ERN and Pe. Gray areas indicated the time range of the Ne/ERN and Pe in each graph. CP = centroparietal, CR = correct response, FC = frontocentral, FE = flanker error, NFG = nonflanker guess, SMI = stimulus‐mask interval. [file PSYP-62-e70128-s002.docx]

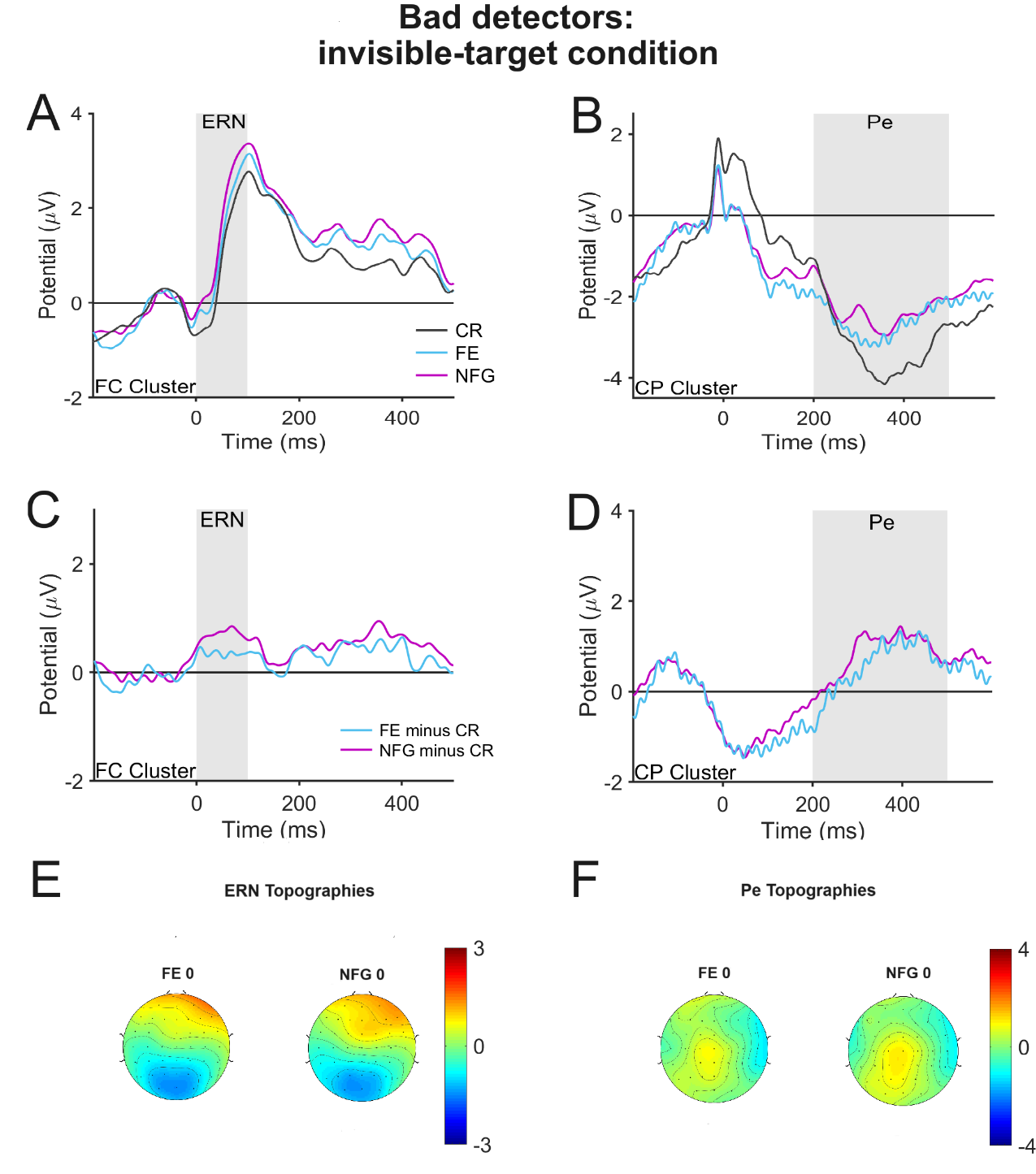


**Figure S5.** Error-related negativity (Ne/ERN; frontocentral cluster in left column) and error positivity (Pe; centroparietal cluster in right column) in the invisible-target (0-SMI) condition of the bad detectors group. All trials were considered irrespective of whether they were correctly classified or not. Data for correct responses were taken from the 133-SMI condition. AB: Waveforms from all response types. CD: Difference waves for flanker errors (minus corrects) and nonflanker guesses (minus corrects). EF: Topographies of the difference waves in the time range of the Ne/ERN and Pe. Grey areas indicated the time range of the Ne/ERN and Pe in each graph. SMI = stimulus-mask interval, CR = correct response, FE = flanker error, NFG = nonflanker guess, FC = frontocentral, CP = centroparietal.
