# Supplementary material for: Error Awareness Can Occur in the Absence of an Error‐Related Negativity
Source: Psychophysiology. 2025 Oct 7;62(10):e70128. doi: 10.1111/psyp.70128 (PMC12504923; doi:10.1111/psyp.70128)
Supplement: Supplementary file 7 — Table S1: Primary task Performance. [file PSYP-62-e70128-s005.docx]

**Table S1.** Primary Task Performance

| **Conditions** | **Error Rates (%)** | **Prop. FE (%)** | **RT Correct (ms)** | **RT FE**  **(ms)** | **RT NFE/G (ms)** |
| --- | --- | --- | --- | --- | --- |
| **Good Detectors** |  |  |  |  |  |
| 250-SMI | 16.0 (±1.7) | 57.2 (±2.3) | 537 (±17) | 531 (±33) | 523 (±31) |
| 133-SMI | 22.5 (±1.6) | 56.8 (±1.5) | 563 (±18) | 551 (±34) | 537 (±27) |
| 0-SMI | - | 31.4 (±1.4) | - | 551 (±22) | 603 (±28) |
| **Bad**  **Detectors** |  |  |  |  |  |
| 250-SMI | 16.4 (±3.1) | 53.9 (±2.3) | 499 (±15) | 492 (±35) | 501 (±48) |
| 133-SMI | 26.9 (±2.7) | 54.9 (±1.4) | 512 (±17) | 479 (±23) | 490 (±25) |
| 0-SMI | - | 38.9 (±3.3) | - | 445 (±16) | 456 (±25) |

*Note.* NFE/G refers to NFE in the 250-SMI and 133-SMI conditions but NFG in the 0-SMI condition. SMI = stimulus-masking interval, ms = milliseconds, RT = response time, FE = flanker error, NFE = nonflanker error, NFG = nonflanker guess, Prop. = proportion. Within-participants standard errors of the mean are provided in parentheses.
