# Supplementary material for: Error Awareness Can Occur in the Absence of an Error‐Related Negativity
Source: Psychophysiology. 2025 Oct 7;62(10):e70128. doi: 10.1111/psyp.70128 (PMC12504923; doi:10.1111/psyp.70128)
Supplement: Supplementary file 8 — Table S2: Frequencies of detection types and trial numbers for flanker errors and nonflanker errors in the visible‐target condition for both subgroups. [file PSYP-62-e70128-s008.docx]

**Table S2**. Frequencies of Detection Types and Trial Numbers for Flanker Errors and Nonflanker Errors in the Visible-Target Condition for Both Subgroups

| **Conditions** | **Frequencies of Detection Types in % and Averaged Trial Numbers** | | |  |
| --- | --- | --- | --- | --- |
|  | **Good Detectors** | **Trials** | **Bad Detectors** | **Trials** |
| **250-SMI** |  |  |  |  |
| **Flanker**  **Errors** |  |  |  |  |
| „correct“ | 4.3 (± 2.1) | 1.2 | 3.2 (± 1.3) | 0.6 |
| „error“ | 88.6 (± 3.1) | 22.2 | 90.8 (± 2.2) | 24.3 |
| „unsure“ | 7.1 (± 1.8) | 1.7 | 6 (± 2.1) | 1.3 |
| **Nonflanker Errors** |  |  |  |  |
| „correct“ | 5.2 (± 2) | 1.3 | 1.5 (± 0.9) | 0.4 |
| „error“ | 89.4 (± 3.2) | 18.4 | 89.8 (± 3.6) | 19 |
| „unsure“ | 5.4 (± 2) | 1.2 | 8.6 (± 3.3) | 1.6 |
|  | **Good Detectors** | **Trials** | **Bad Detectors** | **Trials** |
| **133-SMI** |  |  |  |  |
| **Flanker**  **Errors** |  |  |  |  |
| „correct“ | 10.1 (± 2.8) | 3.4 | 4 (± 1) | 1.8 |
| „error“ | 79.4 (± 3.6) | 29.7 | 89.7 (± 2.5) | 37.7 |
| „unsure“ | 10.5 (± 2) | 4 | 6.2 (± 2) | 2.9 |
| **Nonflanker Errors** |  |  |  |  |
| „correct“ | 5.3 (± 1.3) | 1.5 | 3.4 (± 1.1) | 1.3 |
| „error“ | 82.9 (± 3.2) | 23 | 90.4 (± 2.3) | 31.8 |
| „unsure“ | 11.9 (± 2.3) | 3.4 | 6.2 (± 1.9) | 2.3 |

*Note.* SMI = stimulus-masking interval. Within-participants standard errors of the mean are provided in parentheses.
