# Supplementary material for: Error Awareness Can Occur in the Absence of an Error‐Related Negativity
Source: Psychophysiology. 2025 Oct 7;62(10):e70128. doi: 10.1111/psyp.70128 (PMC12504923; doi:10.1111/psyp.70128)
Supplement: Supplementary file 9 — Table S3: Frequencies of detection types and trial numbers for flanker errors and nonflanker guesses in the invisible‐target condition for both subgroups. [file PSYP-62-e70128-s003.docx]

**Table S3**. Frequencies of Detection Types and Trial Numbers for Flanker Errors and Nonflanker Guesses in the Invisible-Target Condition for Both Subgroups

| **Conditions** | **Frequencies of Detection Types in % and Averaged Trial Numbers** | | |  |
| --- | --- | --- | --- | --- |
|  | **Good Detectors** | **Trials** | **Bad Detectors** | **Trials** |
| **Flanker Errors** |  |  |  |  |
| „correct“ | 0.8 (±0.3) | 0.8 | 11.3 (±7.6) | 20.3 |
| „error“ | 81.6 (±4.3) | 72.8 | 3.9 (±2.3) | 4.8 |
| „unsure“ | 17.7 (±4.2) | 16.8 | 84.9 (±8.4) | 86.8 |
| **Nonflanker Guesses** |  |  |  |  |
| „correct“ | 4.2 (±1.6) | 8.2 | 11.2 (±5.9) | 16.2 |
| „error“ | 2.9 (±0.7) | 5.5 | 5.2 (±3.5) | 5.8 |
| „unsure“ | 93.0 (±1.7) | 184 | 83.6 (±8.2) | 154.2 |

*Note*. Within-participants standard errors of the mean are provided in parentheses.
